# Supplementary material for: Comprehensive exploration of visual working memory mechanisms using large-scale behavioral experiment
Source: Nat Commun. 2025 Feb 5;16:1383. doi: 10.1038/s41467-025-56700-5 (PMC11799313; doi:10.1038/s41467-025-56700-5)
Supplement: Supplementary file 1 — Supplementary Information [file 41467_2025_56700_MOESM1_ESM.pdf]

Supplementary Information

Comprehensive Exploration of Visual Working Memory Mechanisms Using Large-Scale Behavioral Experiment

Liqiang Huang (lqhuang@cuhk.edu.hk)

Department of Psychology, The Chinese University of Hong Kong, Hong Kong, China

Table of Contents

Supplementary Note 1 (Summary of files) ... 4
Supplementary Methods ... 4
Supplementary Methods 1. Information on the method ... 4
Supplementary Methods 1.1. Reasoning behind the chosen methodology ... 4
Supplementary Methods 1.2. Colors ... 5
Supplementary Methods 1.3. Reward to participants ... 6
Supplementary Methods 2. Information on the preliminary analysis ... 6
Supplementary Methods 2.1. Reasoning behind the pattern-level summarization ... 6
Supplementary Methods 2.2. Technical details of preliminary analysis ... 7
Supplementary Methods 3. Information on the neural network ... 7
Supplementary Methods 4. Information on the factorial comparison analysis ... 10
Supplementary Methods 5. Development of the QCE-VWM model ... 11
Supplementary Methods 5.1. Criteria for adding/removing a mechanism ... 11
Supplementary Methods 5.2. Optimization algorithm ... 12
Supplementary Methods 5.3. Scientific regret minimization method ... 13
Supplementary Methods 5.4. Iterative refinement of the model ... 14
Supplementary Discussion ... 16
Supplementary Discussion 1. Step-by-step explanation of the QCE-VWM model ... 16
Step 1a. Interactions between items ... 18
Step 1b. Chunking ... 18
Step 2a. Eight color categories ... 19
Step 2b. Weights of color-category-biased components ... 20
Step 2c. Weights of unbiased component ... 20
Step 2d. Concentration ... 20
Step 2e. Crosstalk ... 21
Step 2f. Weights of Swap-based components ... 21
Step 2g. Weights of all 3 types of components ... 21
Step 3a. Attraction toward centers ... 22
Step 3b. Biases of distributions ... 22

Step 3c. SDs of distributions 22
Step 3d. Retention rates of items 23
Step 3e. Trade-off 23
Step 3f. Distribution of responses (with low-precision components) 23
Supplementary Discussion 2. Statistical evidence for the QCE-VWM model 24
Supplementary Discussion 2.1. The 17 alternative models 24
Supplementary Discussion 2.2. Scripts of the alternative models 25
Supplementary Discussion 2.3. Model comparison 26
Supplementary Discussion 2.4. Cross-validation 27
Supplementary Discussion 3. Other analysis and discussion 28
Supplementary Discussion 3.1. Spatial attention 28
Supplementary Discussion 3.2. Low-precision component 29
Supplementary Discussion 3.3. Slot vs. Resource 30
Supplementary Discussion 3.4. Color categories 30
Supplementary Discussion 3.5. Advantage and disadvantage of reddish colors 31
Supplementary Discussion 3.6. Deviations from Bayesian principles 32
Supplementary Discussion 3.7. Early vs. late processing 33
Supplementary Discussion 3.8. Spatial binding errors 34
Supplementary Discussion 3.9. How items affect each other 34
Supplementary Discussion 3.10. Normal distribution in circular space 35
Supplementary Discussion 3.11. Order of report 37
Supplementary Discussion 3.12. Age and gender 38
References in the Supplementary Information 39

Supplementary Note 1 (Summary of files)

All the data and scripts used for analysis will be made available on the Open Science Framework upon formal acceptance of this paper for publication. These files are organized into six folders:

- The “data” folder contains all the data files (.mat or .csv) discussed in this study.
- The “QCE_VWM” folder contains MATLAB scripts (run on R2022b) used for the QCE-VWM model or related analysis (e.g., alternative models). Below, when a MATLAB script (.m) is discussed, it can be found in this folder unless otherwise specified.
- The “images” folder contains 10,000 PNG images, each visually illustrating the information of a pattern, including the colors used, the distribution of responses, and predictions made by the QCE_VWM model (illustrated by a white curve) and the guidance neural network (black curve).
- The “factorial”, “preliminary” and “neural_network” folders will be discussed in the relevant sections.

Supplementary Methods

Supplementary Methods 1. Information on the method

Supplementary Methods 1.1. Reasoning behind the chosen methodology

The current working memory task always involves memorizing four colors. This differs from most previous studies which varied the number of items to be memorized, typically ranging from 1 to 6 or 8. The rationale behind this “fixed set size” is that the number of items has a dominant effect on performance, a well-established and, therefore, non-informative phenomenon. Consequently, the set size (i.e., the number of items) is kept constant, so the data can focus on aspects that are less understood, such as how multiple colors are memorized together.

As for the reason behind fixing the set size at four, on the one hand, the number 4 was chosen over smaller numbers to potentially shed light on the slot/resource debate. According to Zhang and Luck (2008), approximately 3 colors can be retained without resorting to random guesses. Thus, using 3 or fewer colors would likely make the results ineffective in distinguishing between slot and resource models. On the other hand, the number 4 was selected over larger numbers to minimize the impact of forgetting, which could become significant after several responses have been made.

The participants were required to respond to all four colors in each trial, thus yielding more information for the study.

The effects of spatial binding errors were expected and indeed played a significant role (see Supplementary Discussion 3.8). Nevertheless, to minimize spatial binding errors, the four colored squares were always presented at the four corners to facilitate easier recollection of their locations.

Previous studies on this topic sometimes implemented verbal suppression to minimize the influence of verbal coding. However, this was not implemented in the current study because earlier research

indicated that color categories play roles in VWM that extend well beyond mere verbal codes (Bae et al, 2015). Moreover, for the current aim of a comprehensive exploration, it's preferable to include all relevant mechanisms.

Supplementary Methods 1.2. Colors

The colors used in the present study were selected from a set of 360 colors borrowed from a previous study by Adam, Vogel, and Awh (2017). The RGB values of these 360 colors were downloaded from https://osf.io/vzmd6. In the study by Adam, Vogel, and Awh (2017), these colors were distributed along a circle within the CIE-Lab color space, with each color being identified by its corresponding angle on this circle, ranging from 0° to 359°. Unless specified otherwise, these angles are expressed in degrees, not radians.

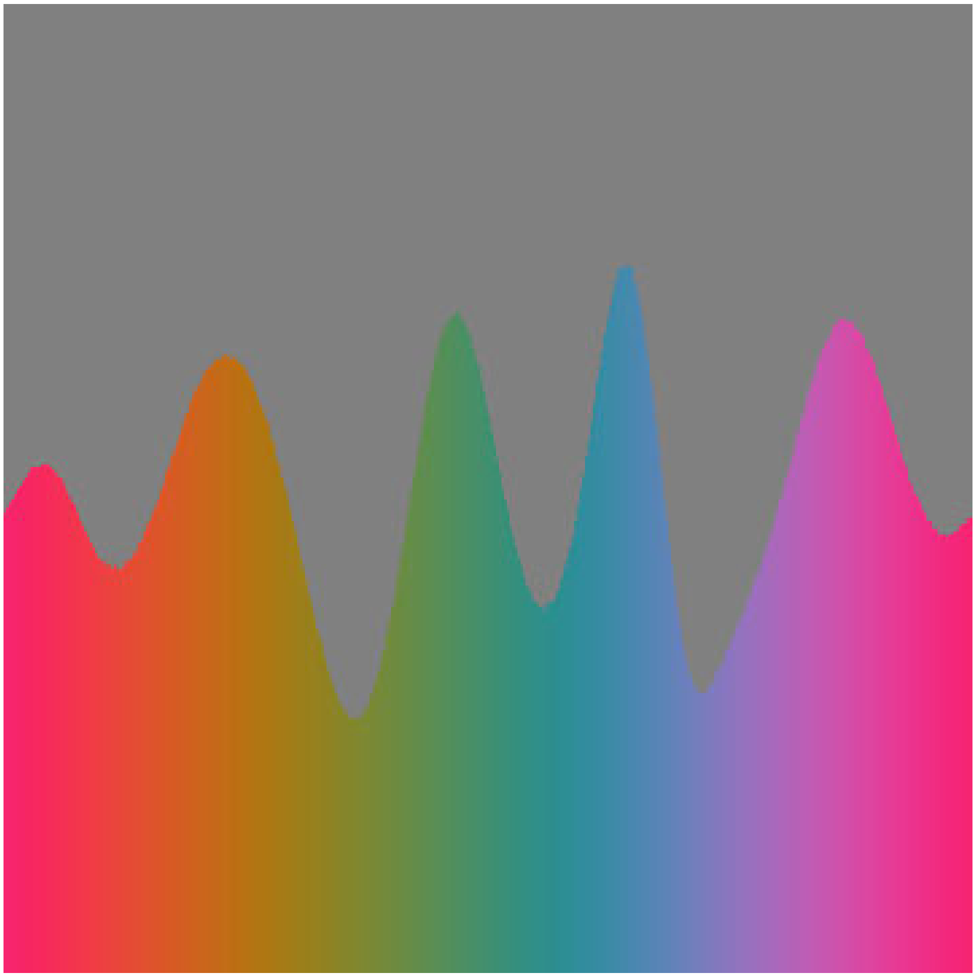

Supplementary Figure 1. Distribution of responses. The responses to colors are highly uneven on the color wheel, clustering around the major categories "red," "yellow," "green," "blue," and "purple." The presence of these peaks suggests that the participants of this study have seen the colors reasonably consistently.

In the present study, participants used their own devices, the actual colors displayed inevitably varied slightly between devices, implying slight inconsistencies from their designed values in the CIE-Lab color space. To assess the degree to which these inconsistencies might have affected the results, the overall distribution of responses was inspected. Specifically, as Bae et al (2015) showed in their Fig 6b, the responses clustered around the category centers, with a few obvious peaks. In the present study, if the different devices had displayed the colors in rather different ways, then different participants' peaks would fall in different positions on the color wheel and would smooth out. As shown in Supplementary Figure 1, the peaks in the distribution of responses in the present study are clear, with amplitudes comparable to those in Fig 6b of Bae et al (2015). Therefore, it seems that the participants of this study have seen the colors reasonably consistently, and the slight inconsistencies in the actual colors they saw are unlikely to have greatly affected the current results.

The 10,000 color patterns are stored in the file "patterns.mat", which is a 10,000 rows × 4 columns matrix filled with random integers in the range of [0, 359].

Supplementary Methods 1.3. Reward to participants

Rewards for participants were primarily based on their level of participation, supplemented by a lottery system. Regarding participation, participants received one credit upon completing a block of 10 trials, provided that the average root mean square error (RMSE) in their responses, compared to the actual colors, was less than 90°. Each block took approximately two minutes. They were subsequently ranked based on their cumulative credits. At the conclusion of each week-long session, a cutoff point was determined based on the cumulative credits of the participant ranked 200th among all participants. Those with credits surpassing 75% of this cutoff point, typically encompassing 300-400 participants, received a prize of 25 Chinese Yuan.

As for the lottery system, during each week-long session, two participants were randomly chosen from the top 200 to receive special prizes, with each prize valued at 500 Chinese Yuan.

Supplementary Methods 2. Information on the preliminary analysis

Supplementary Methods 2.1. Reasoning behind the pattern-level summarization

As stated in the main text, the data analysis of this study focuses on a pattern-level summary (i.e., distribution of responses). This is because this level of summarization offers a suitable level of complexity (40,000 360-degree distributions): intricate enough to be richly informative, yet not too complex to be unmanageable.

On the one hand, opting for a much more simplified analysis, such as dividing the data into a few conditions based on certain hypotheses, would resemble a typical experimental study and fail to fully utilize the rich information provided by the large-scale experiment.

On the other hand, choosing a much more detailed analysis, like treating all 10 million trials individually and attempting to find the model that provides the best predictions for memory performance at the trial level, would certainly capture more useful mechanisms (e.g., cross-participant variability in

precision, serial dependence, order of report, interference across trials in a run) than the current QCE-VWM model. However, this approach would far exceed the practical limits of a study. Specifically, the development of the current QCE-VWM model took approximately eight months (see Supplementary Methods 5.4 for more details). A model at the trial level would need to handle data in much more detail and would take significantly longer to optimize. It would also introduce additional factors, leading to a greater number of possible models. All things considered, a model at the trial level is clearly impractical.

Some of the most critical trial-level information, including the effects of the order of report and the influences of age and gender, has been analyzed separately and is available in the Supplementary Discussion 3.11 and Supplementary Discussion 3.12.

Supplementary Methods 2.2. Technical details of preliminary analysis

The raw data used in this study is saved in the file "raw_data.csv". All the data are collected through our laboratory's online platform (https://huang.psy.cuhk.edu.hk/games/). This platform is specifically designed for WeChat users. To enhance the user experience, this webpage automatically detects WeChat IDs and can only be accessed within the WeChat app. To try it out, please open the webpage using the WeChat app.

The "raw_data.csv" file consists of two columns × 1,015,925 rows, each rows represents the data of a 10-trial session. The first column "ID" shows the participant for each session. For privacy, the ID displayed here is a unique internal identifier and not the same as the ID on the participant's screen, but it maps one-to-one to it. The second column "data" shows the data for each session, which contains a total of 90 numbers. The first 10 numbers shows the 10 patterns used in the session, whereas the rest are 40 pairs that are separated by "/". Each pair displays the response given to each color item and its corresponding response time (in 0.1-second units). No analysis was conducted on the response times.

The script "preliminary/raw_data_to_formatted_data.m" converts raw data from "raw_data.xlsx" to "formatted_data.mat" and takes up to an hour to run.

The script "preliminary/formatted_data_to_distribution.m" processes formatted data to calculates the distribution of responses by summing up the responses of all trials using each pattern. As discussed in the main text, some data were excluded in this process.

These distributions of responses are saved in two files. The file "distribution_data.mat" was used by the QCE-VWM model and it consists of a 40,000 rows × 360 columns matrix. Each row represents the distribution of responses made to one of the 40,000 colors in the 10,000 patterns. The same set of data is reformatted differently in the file "data_neural_network.csv" for more convenient use by the neural networks. Specifically, the 4 distributions of responses to the 4 colors in each pattern were merged in one row. In addition, each of the 4 colors of each pattern was represented as a pair of numbers showing the x/y coordinates of that color on a color wheel. Please refer to Supplementary Methods 3 for details on how these data are utilized in the neural networks.

Supplementary Methods 3. Information on the neural network

The CNN network implemented in the script "neural_network/neural_network.py" was run on PyTorch version 1.12.0.

As discussed in the main text, the NLLsr loss function is the negative log-likelihood of a single response. Each response is compared to the neural networks' predicted distribution of responses to calculate the NLLsr. The average NLLsr of all responses of all trials is then calculated to evaluate the overall fit of the neural networks.

The NLL loss function was chosen over other potential indexes, such as the root mean square error (RMSE), because it provides a better measure of the amount of information in the responses. For modeling purposes, NLLsr and NLL_{total} are equivalent. However, the former is chosen since the value of the latter varies proportionally with the volume of data, which can create confusions in certain scenarios, such as comparing a model trained on the whole dataset with another utilizing only part of the data.

The Adam optimizer was used.

A validation-based early stopping strategy was implemented to prevent overfitting. The dataset was randomly divided into training and validation sets, with 5,000 patterns in each set. The neural networks was trained on the training set and the average NLLsr of the validation set was calculated after every 100 epochs. The training was terminated when the NLLsr increased from the previous evaluation. Then, the roles of the training and validation sets were swapped, and the same procedure was repeated.

As explained in Supplementary Methods 1.2, each color in this study is described by an angle on a color wheel, varying across 360 discrete levels (i.e., 0~359°). In current neural networks, each color is represented by a pair of numbers that denote the x/y coordinates (i.e., Cartesian coordinates) on the color wheel, corresponding to the angle of that color (i.e., the angular coordinate in Polar coordinates). This angular-to-Cartesian conversion is conducted as [x, y] = [cos(angle), sin(angle)]. This angular-to-Cartesian conversion is necessary because the x/y coordinates inherently reflect the cyclic nature of the color wheel within the neural networks, whereas the angles do not. For example, the colors represented by 1° and 359° are very similar to each other. This similarity is not directly reflected by the angles themselves but is clearly represented in the x/y coordinates. A comparison conducted between the current neural network and another neural network that directly uses angles to represent colors has confirmed that converting from angles to x/y coordinates indeed improves the performance of the neural network. In total, the present neural networks take eight values as the input, namely x/y coordinates of the four colors of each pattern.

As explained in the main text, the output layer's 196 neurons are designed to represent observers' responses by mixing 16 normally distributed components—representing knowledge-based responses—with a fraction of random guesses. Specifically, each set of three values is designed to delineate the center, amplitude, and standard deviation (SD) of a component's distribution, which totals 3 × 16 components × 4 colors = 192 values. The remaining four values are tailored to depict the fraction of random guesses for each of the four colors.

Following common practice in neural networking, the optimal complexity of the network was determined through validation. Each setting was tested 12 times. As shown in Supplementary Figure 2,

for fitting the validation data, it was optimal to have 100 neurons in the intermediate layers and 16 components in knowledge-based responses.

This specific neural network (100 neurons and 16 components) was used to guide the development of the QCE-VWM model and is referred to as the "guidance neural network".

To determine whether the complexity of the guidance neural network is essential, or in other words, whether it can be significantly simplified without a substantial decrease in data fitting, four reduced versions of the guidance neural network were tested. These versions respectively possess 8,100 parameters (50 neurons & 8 components), 2,227 parameters (25 neurons & 4 components), 628 parameters (12 neurons & 2 components), and 208 parameters (6 neurons & 1 component). As illustrated in Figure 8b of the main text, the performance of the neural network significantly decreases when its parameter count is reduced to 628 and 208. This confirms that the neural network cannot remain effective without its complexity, highlighting the distinct advantage of the QCE-VWM model in simultaneously achieving effectiveness and parsimony.

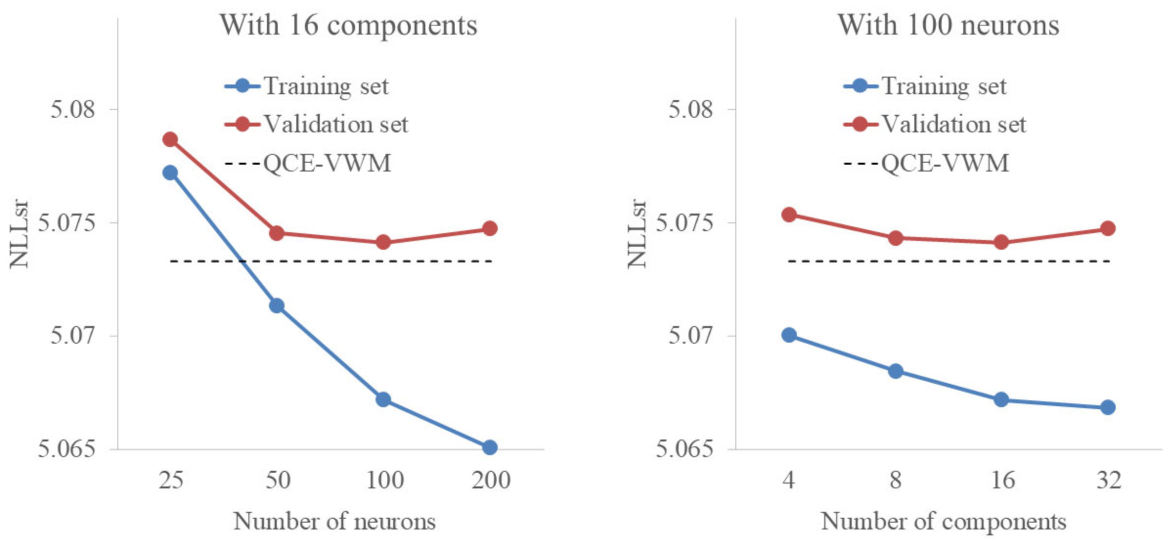

Supplementary Figure 2. Optimal Complexity of the Neural Network. The left panel shows how the NLLsr loss function varies with the number of neurons in the intermediate layers. For fitting the validation data, it was optimal to have 100 neurons. Both simpler and more complex models resulted in a decline in the fit to the validation data. Similarly, the right panel indicates that it was optimal to have 16 components in knowledge-based responses. The dashed line represents the NLLsr of the QCE-VWM model, which is presented here for comparison. It outperforms the NLLsr in the validation sets of all neural networks.

In addition to this particular approach (i.e., simulating the distribution of responses as a mixture of knowledge-based components and random guesses), several attempts were made using other methods. For instance, neural networks were trained to directly predict the distribution of responses. However, these approaches have proven less effective in fitting the validation data.

Supplementary Methods 4. Information on the factorial comparison analysis

The study by Van den Berg, Awh, and Ma (2014) provides a comprehensive comparison of models of visual working memory, focusing on three key questions: the nature of mnemonic precision, the number of items remembered, and the role of spatial binding errors.

Regarding the nature of mnemonic precision, Van den Berg, Awh, and Ma (2014) distinguished four possibilities: the FP (Fixed Precision) condition, where the precision of a remembered item is fixed across items, trials, and set sizes; the SA (Slots Plus Averaging) condition, where the precision of a remembered item is provided by discrete slots and is thus quantized; the EP (Equal Precision) condition, where the precision of a remembered item is equal across items and trials but depends in a power-law fashion on set size; and the VP (Variable Precision) condition, where the precision of a remembered item varies across items and trials. In the present study, the set size is fixed at four. Therefore, the SA and EP conditions are indistinguishable from the FP, leaving only the FP and VP conditions for comparison.

Regarding the number of items remembered, Van den Berg, Awh, and Ma (2014) distinguished four possibilities: the A condition, where all items are remembered; the F condition, where there is a fixed number of remembered items; the P condition, where the number of remembered items varies across trials and follows a Poisson distribution; and the U condition, where the number of remembered items varies across trials and follows a uniform distribution. In the present study, only the pattern-level summary is analyzed, focusing on how many items are remembered on average across all relevant trials. Therefore, the P and U conditions are indistinguishable from the F, leading to a comparison of only the A and F conditions.

On the role of spatial binding errors, Van den Berg, Awh, and Ma (2014) used NT to indicate the presence of non-target responses.

To summarize, the present study conducted a factorial comparison on the same three questions raised by Van den Berg, Awh, and Ma (2014). After excluding some inapplicable conditions, we have two levels of precision (FP vs. VP), two levels of the number of items (A vs. F), and two levels of spatial binding errors (NT vs. no NT), resulting in a total of $2\times2\times2 = 8$ possible models.

This factorial comparison was implemented using a set of scripts in the “factorial” folder. Aside from “Factorial_model.m”, all other scripts are similar to their counterparts in the “QCE-VWM” folder and will not be elaborated upon.

All three aspects are manipulated in “Factorial_model.m”. A total of five parameters are used: the parameter “FP_precision” specifies the precision in the FP condition, while the parameters “VP_gamma_k” and “VP_gamma_theta” define the gamma distribution in the VP condition. The

parameter “F_n_item_remembered” specifies the number of items remembered in the F condition. The parameter “NT_p_NT” is used to specify the proportion of non-target responses in the NT condition.

As shown in Figure 9 of the main text, the VP-F-NT model is the best model among all eight models considered, which is consistent with the findings of Van den Berg, Awh, and Ma (2014). The best fitting parameters for this VP-F-NT model are as follows. The F_n_item_remembered (number of remembered items) is 3.49. The NT_p_NT” (proportion of non-target responses) is 2.47%. The values “VP_gamma_k” and “VP_gamma_theta” are 1.916 and 0.000988, respectively, implying that the median SD is 24°, and 90% of the SDs are distributed in the range of [14.0°, 53.5°].

Supplementary Methods 5. Development of the QCE-VWM model

The QCE-VWM model is implemented in the “QCE_VWM.m” function. To see the output of the model, set the “i_model” as “1” and run the script “show_model_results.m”.

As stated in the main text, the QCE-VWM model was developed by integrating existing concepts from the literature and employing the scientific regret minimization approach to pinpoint potential mechanisms.

Supplementary Methods 5.1. Criteria for adding/removing a mechanism

A single “mechanism” within the QCE-VWM model refers to a group of calculations centered around a concept. It is important to clarify that, although a mechanism can sometimes represent a step (for example, interactions between items, chunking, trade-off, concentration, crosstalk), mechanisms and steps are not always synonymous. For instance, the “unequal spatial attention” mechanism encompasses all calculations related to spatial inhomogeneity, which are distributed across various steps.

The decision to retain a mechanism is based on both its conceptual plausibility and a statistical index I have created, named CAD (complexity-adjusted d). This index is based on Cohen’s d but adjusts for a fair comparison between simpler and more complex mechanisms. For example, eliminating the chunking mechanism results in a reduction of 3 parameters, whereas eliminating the trade-off mechanism results in a reduction of only 1 parameter. Therefore, the CAD is necessary for a fair comparison in these two situations.

Specifically, the importance of a mechanism is assessed using the CAD of the comparison between two models: one that incorporates the mechanism and one that does not. Both models are first optimized, and the benefit of including the mechanism is then quantified as the reduction in NLLsr, evaluated across all 10,000 patterns. A t-test is then applied to assess the consistency of these benefits across these 10,000 patterns. The effect size, or Cohen’s d value, derived from this t-test, is subsequently adjusted for the mechanism’s complexity. This adjustment involves dividing Cohen’s d by the square root of Δ_{param} (i.e., the difference in the number of parameters between the two models). For the Δ_{param} values involved in this study, see Supplementary Table 1.

$$CAD \text{ (complexity adjusted d)} = \frac{Cohen's\ d}{\sqrt{\Delta_{param}}}$$

The most significant advantage of CAD is its direct relation to Cohen's d, which facilitates easy comprehension. In terms of correcting for the effect of complexity, there is no established standard for addressing this particular situation; thus, the approach is based on the following simplified logic. If N independent variables each have a mean of m and a standard deviation of sd, the expected effect size of each variable is m/sd. The effect size of their sum is the expected mean ($N \times m$) divided by the expected standard deviation ($sqrt(N) \times sd$), simplifying to $sqrt(N) \times m/sd$. To generalize, the effect size for the sum of several variables is anticipated to increase proportionally with the square root of the number of variables[1]. Therefore, the CAD is calculated by dividing Cohen's d by the square root of Δ_{param} here.

Having introduced the CAD, the criterion used for retaining a mechanism in this study is a minimum CAD of 0.2. This criterion is chosen because a Cohen's d of 0.2 is generally considered a small but acceptable effect size in experimental psychology.

This criterion (CAD > 0.2) is not intended to be a rigid standard and should be adjusted based on conceptual interpretations in future studies. For example, if mechanisms generally have smaller CADs but are conceptually appealing, the criterion might be lowered. Conversely, it could be raised for mechanisms that generally have larger CADs.

For the CAD values in comparing the QCE-VWM and 17 alternative models, please refer to Supplementary Table 3. For the reasons why this criterion is based on Cohen's d in an analysis of consistency across patterns, please see Supplementary Discussion 2.3.

Supplementary Methods 5.2. Optimization algorithm

The optimal values for the parameters of the QCE-VWM model were determined through an optimization algorithm. Specifically, a random search optimization algorithm was chosen for its general applicability to the wide range of mechanisms in the QCE-VWM model. While this algorithm may not be the most efficient, it allows the model to be optimized without relying on gradient-based techniques, which can be infeasible in some cases.

Like the guidance neural network, this optimization algorithm maximized the likelihood of the data by using the NLLsr loss function. The optimization procedure was performed using two functions that respectively ran mini-batch and whole-set optimization. Each function consisted of multiple steps, during which the program randomly selected a parameter and adjusted it in a random direction by one step. If the new value resulted in a smaller NLLsr, it was accepted.

The mini-batch optimization process was performed using the "optimization_batch.m" function. This function split the data into 10 mini-batches. For each batch, optimization was performed for 20 steps before moving on to the next batch. This process was repeated for five epochs, so a total of 1,000

[1] The variables of a mechanism may not adhere to these ideal assumptions, and they will be penalized in such situations. This is not a deficiency of the CAD index; rather, it is intentional because the CAD is designed to assess the degree of enhancement on the overall model. For example, if the effects of several variables are redundant, their combined effect will be smaller than what the "square root relationship" predicts. In a way, the CAD underestimates the effect size of each individual variable, but this underestimation is exactly what is necessary to penalize the redundancy among these variables.

steps were taken (20 steps × 10 batches × 5 epochs). For each parameter, the step size was adjusted with the goal that the "intended effect of each step" is 0.0001. Specifically, the step size increases if the effect of a step is smaller than 0.0001 and decreases if it is greater than 0.0001.

The whole-set optimization process was conducted using the "optimization_whole_set.m" function. This function utilized the entire dataset for optimization, which consisted of multiple rounds. In each round, each parameter was adjusted until a local minimum was reached, and the parameters were adjusted in a random order. The optimization continued until the improvement of NLLsr in a round was less than one ten-millionth (0.0000001). Here, the step size was also subject to the same type of adjustment as implemented in the mini-batch optimization, but now the "intended effect of each step" is 0.000001.

The optimization of the model was performed using the "optimization.m" script. This script repeated the mini-batch optimization process until the best-fitting NLLsr was not improved for 10 consecutive runs. Then, it ran the whole-set optimization process once. Both the mini-batch and the whole-set optimization used the best-fitting parameters from the previous runs as the starting values of the parameters. In mini-batch optimization, random noise was added to the starting values half the time if the NLLsr did not improve in the previous run. This random noise aided in preventing the model from becoming trapped in a local minimum.

After the whole optimization process was completed, the optimized parameters were manually saved in the "saved_parameters.m" script.

The models were developed gradually. When a new model was optimized, the starting values of the parameters were chosen based on their optimized values in the previous models. For parameters that were added for the first time, the starting value was set according to conceptual interpretations.

Supplementary Methods 5.3. Scientific regret minimization method

As discussed in the main text, the current study adopts the scientific regret minimization method (Agrawal, Peterson, & Griffiths, 2020) and utilizes the predictions produced by the guidance neural network, rather than the actual data, as the guidance for model development. Specifically, the predictions of the under-development QCE-VWM model are compared with those of the guidance neural network to identify what is lacking in the former.

The scientific regret minimization method (Agrawal, Peterson, & Griffiths, 2020) is mainly used in two ways in the present study. A first application is the visualization of the worst-fitted patterns. Specifically, whenever the QCE-VWM model is updated, the 500 worst-fitted patterns (i.e., those in which the QCE-VWM underperforms the guidance neural network the most) are visualized in the style of Figure 2 of the main text, allowing us to see what is missing in the former.

A second application of the scientific regret minimization method is generating predictions of out-of-dataset patterns for a full-range characterization of a mechanism. For instance, to understand how the precision and bias of an item's distribution of responses are affected by other items, it would be ideal to have data for all possible patterns. This would allow us to directly observe how an item's precision and bias change when another item shifts on the color wheel. However, although the present study has

utilized an unusually large set of 10,000 patterns, this represents only a minuscule fraction of all the 360^4 = 16,796,160,000 possible patterns. Consequently, we can only find scattered examples and are left to infer what might happen in the gaps between these examples. Now, by utilizing the scientific regret minimization method, we can leverage the guidance neural network to generate virtual data (i.e., predictions from the guidance neural network) for all necessary patterns. This approach allows us to observe how an item's precision and bias change when another item shifts on the color wheel. In addition, we can undertake virtual experiments; for instance, we could choose a set of 10,000 patterns based on the theoretical hypothesis we aim to explore and summarize the virtual findings.

Of course, we can combine the two applications. Namely, we can simultaneously produce predictions from both the guidance neural network and the QCE-VWM model for a set of patterns chosen for a theoretical hypothesis, and see when and how their predictions diverge from one another.

One surprising outcome of this study is that the QCE-VWM model, despite being guided by the neural network, has eventually outperformed it in terms of data fit. How is this possible? As mentioned in the main text, the QCE-VWM model, while guided by the neural network, is always fit to the actual data. Therefore, it is technically possible for the outcome of this guidance to surpass the guidance itself. This is because neural networks, by their nature, ensure that their predictions are never perfect. They may come very close to the truth but are always slightly off from the exact truth. However, when used in conjunction with conceptual judgment, they may lead us to the exact truth.

Supplementary Methods 5.4. Iterative refinement of the model

As illustrated in Figure 1c of the main text, the QCE-VWM model was iteratively refined to determine which mechanisms should be included and how they should be assembled. Conceptually, this iterative refinement is similar to a manually-executed step-wise regression. Using the aforementioned criterion (conceptually plausible and CAD > 0.2), a candidate mechanism meeting this criterion is added to the model, while one that no longer meets this criterion is dropped from the model. The entire process of iterative refinement was guided by insights from previous studies (see Table 1) and those from the scientific regret minimization method (see Supplementary Methods 5.3). Below is a sketch of the actual iterative refinement process in this study:

First, the QCE-VWM model was created by combining color categories with factors considered in the slot/resource debate, such as random guesses, variability in the precision of representations, and spatial binding errors. Several different approaches were tried, and it was found best to characterize the roles of color categories and spatial binding errors both as components (i.e., color-category-biased components and swap-based component) separate from a component representing the precise color value (i.e., unbiased component). In this process, the variability in precision was simplified to a single low-precision component for practical reasons (see Supplementary Discussion 3.2), and strict Bayesian integration was found to be unsatisfactory.

Second, several well-established mechanisms, such as interactions between items, chunking, and trade-off, were added to the model and tested to determine their relationships with other mechanisms.

Third, an attempt was made to explore the conjunctions between the three important conceptual aspects of the model: category, interaction, and guess. As discussed in the main text, the conjunction between “category” and “interaction” (i.e., how the weights of items affect each other) led to the discovery of two new mechanisms: concentration and crosstalk. The conjunction between “category” and “guess” (i.e., are the chance of being remembered influenced by categories?) led to the use of categories in calculating the retention rate. The conjunction between “interaction” and “guess” (i.e., how items affect each other’s chance of being remembered) led to the “interaction on retention” effect in step 1a.

Fourth, spatial inhomogeneity in retention rates, presumably caused by unequal spatial attention, was noticed and subsequently tested on other mechanisms.

This iterative refinement is inevitably partly subjective. Although an objective criterion (CAD > 0.2) is observed to ensure that all adopted mechanisms must substantially contribute to the model, many decisions (e.g., what are the plausible mechanisms from the literature and from the observation of data, how different mechanisms should be assembled) inevitably depend on the researcher’s subjective judgement. Therefore, as stated in the main text, the comprehensive exploration approach is iterative in nature. The current version of the QCE-VWM cannot be guaranteed to be the globally optimal model. However, this is a necessary cost for the greater benefit of exploring an unlimited solution space, thereby enabling the creation of a more effective model.

To mitigate the limitations of subjective judgment and to find a model that is as good as possible, the model development process explores options as exhaustively as possible at all levels. This exhaustiveness has proven beneficial in a number of instances. Below are a few examples:

First, regarding the relations between mechanisms, the interactions between items and chunking were tested at both the pre-categorical and category-based information levels, even though I initially speculated that category-based was more plausible than pre-categorical. However, contrary to my initial speculation, the pre-categorical level turned out to be the better solution (see Supplementary Discussion 3.7 for details).

Second, concerning the possible implementations of mechanisms, spatial binding errors were examined in both the representation stage (i.e., swap-based components) and the response stage. Despite my initial belief that the response stage was more plausible than the representation stage, the latter proved to be the better solution (see Supplementary Discussion 3.8 for details).

Third, in terms of within-mechanism details, the initial exploration of a mechanism always begins with four sub-components, each equipped with a separate set of parameters to allow them to exhibit distinct characteristics during optimization. These sub-components are prepared to account for intricate sub-mechanisms and often turn out to be redundant, subsequently being simplified. But in one case, this does lead to discovery of distinctive sub-mechanisms. Specifically, in exploring the conjunction between “category” and “interaction” (i.e., how the weights of items affect each other), three sub-components were found to be reliable and distinct from each other. Two of these three are unaffected by the relative magnitude of the weights and affect different ranges of color differences; they were merged to become

the "crosstalk" mechanism. The third is affected by the relative magnitude of the weights and become the "concentration" mechanism.

As a result of exhaustive exploration, approximately one thousand candidate models were tested during the iterative refinement of the QCE-VWM model[2]. The optimization of each model usually takes 6 to 10 hours to complete, so the entire model development process spanned approximately eight months.

Supplementary Discussion

Supplementary Discussion 1. Step-by-step explanation of the QCE-VWM model

Before delving into a detailed explanation of the QCE-VWM model, several clarifications are necessary.

First, this model simulates observers' responses by melding normally distributed components, which depict knowledge-based responses, with a segment of random guesses. The proportion of the knowledge-based responses is termed the "retention rate".

Second, each normally distributed component is defined by its mean and SD. The manner in which these attributes are influenced by various factors will be outlined in the following discussion. It's worth noting that these normally distributed components exist within a circular space, and are implemented as the truncated normal distribution (see Supplementary Discussion 3.10).

Third, the random guess is defined as a uniform distribution over the 360° range.

Fourth, in the subsequent description of the steps, parameters are denoted with the notation $P_{parameter\_name}$. All 57 parameters, distributed across 27 variables, are listed in Supplementary Table 1. All other variables in the script use the notation $V_{variable\_name}$.

Fifth, as previously stated, each color is characterized by its corresponding angle on a circle. Additionally, the difference between two colors is represented simply by their angular difference.

Sixth, the items 1-4 respectively refer to the items on the top-left, bottom-left, top-right, and bottom-right corners.

Seventh, in the present study, the precision of representation is primarily expressed as SDs. They can be converted to each other using the formula: $Precision = 1/SD^2$.

---

[2] In this case, a potential concern is the multiple testing problem. In the fields of statistics and modeling, the "multiple testing problem" refers to the issue that arises when conducting a large number of statistical tests: the probability of obtaining a significant result purely by chance (a false positive) increases. When addressing the multiple testing problem, a critical metric is the product of the significance level (p) and the number of tests conducted. For instance, with a p-value of 0.05 and 1,000 tests, approximately 50 false positives would be expected purely by chance, assuming all null hypotheses are true and no real effects exist. However, as shown in Supplementary Table 3, all the p-values in the comparisons conducted in the present study are extremely small, suggesting that the multiple testing problem is negligible in this context.

Step		Name	Index	Number of irrelevant parameters in the alternative model														
				2	3	4	5	6	7	8	9	10	11	12	13	14	15	16
1	a	general_SD	1-2	1														
		interaction_on_retention_magnitude	3	1									1					
		interaction_on_bias_magnitude	4-5	2														
	b	utility_of_chunk_structure_strength	6		1													
2	a	center_of_8_color_category	7-13			2	1											
		log_SD_of_8_color_category	14-20			2	1											
		log_amplitude_of_8_color_category	21-27			2	1											
		log_attraction_of_8_color_category	28-35			2	1											8
		spatial_attention_on_variable	36-37														2	
	c	spatial_attention_on_unbiased	38						1								1	
	d	concentration_magnitude	39								1							
	e	crosstalk_magnitude	40									1						
		crosstalk_SD	41									1						
	f	swap_weight_SD	42							1								
		swap_weight_amplitude	43							1								
		chunking_on_swap	44		1					1								
3	a	chunking_on_attraction	45		1													
	c	SD_ratio_component_to_weight	46															
		red_advantage_as_category	47					1										
		log_SD_unbiased_baseline	48						1									
		red_disadvantage_on_unbiased	49						1							1		
	d	retention_weight_p	50-52										3					
		retention_baseline	53										1					
		spatial_attention_on_retention	54										1				1	
	e	trade_off_effect	55										1	1				
	f	low_precision_portion	56												1			
		low_precision_SD_ratio	57												1			
		Total number of irrelevant parameters		4	3	8	4	1	3	3	1	2	7	1	2	1	4	8
		Number of newly added parameters																1
		Δparam = difference between the two rows above		4	3	8	4	1	3	3	1	2	7	1	2	1	4	7

Supplementary Table 1. A list of the parameters. This table lists all 57 parameters represented across 27 variables. For details on each parameter, please refer to Supplementary Discussion 1. The table also presents Δparam, which denotes the number of parameters rendered irrelevant in each of 15 alternative models (models 2-16). Δparam is used to calculate the CAD, a major statistical index in this study (the Supplementary Methods 5.1). All parameters are relevant in models 17-18, hence they are not included here. It should be noted that model 16 introduces a new parameter (data_SD), used to specify the SD of data in Bayesian integration. Although this new parameter occupies the 28th position in the array, it is irrelevant to the parameter that typically occupies this position (i.e., $P_{log\_attraction\_of\_8\_color\_category}$).

Eighth, the model's script can automatically adjust to the size of the data. However, for simplicity in the explanations, it is assumed that the whole dataset (10,000 patterns or 40,000 items) is being processed.

Step 1a. Interactions between items

In the QCE-VWM model, the interactions between items influence the retention rates of the items and introduce biases to their memorized colors. These two effects are denoted as $V_{interaction\_on\_retention}$ and $V_{interaction\_on\_bias}$, respectively, and will be utilized in subsequent steps.

Both $V_{interaction\_on\_retention}$ and $V_{interaction\_on\_bias}$ are computed by aggregating the mutual impacts of all items. The influence of each item on another is a function of $V_{color\_difference}$, which indicates the distance between them on the color wheel. The latter function ($V_{interaction\_on\_bias\_function}$) includes a positive short-range component and a negative long-range component, as shown in Figure 5b of the main text. The magnitudes of these two components are specified by $P_{interaction\_on\_bias\_magnitude}$. Conversely, the former function ($V_{interaction\_on\_retention\_function}$) only includes the positive short-range component, as depicted in Figure 5a of the main text, with its magnitude specified by $P_{interaction\_on\_retention\_magnitude}$. These components are modeled as normal distributions, with their SDs defined by the parameter $P_{general\_SD}$.

Step 1b. Chunking

In the QCE-VWM model, the overall chunking effect of a pattern is calculated as a "weighted average". The elements being averaged are the chunking effects for all possible chunking structures, whereas the weights used for averaging are determined by the likelihood of each structure.

All the 15 possible chunking structures are outlined in Supplementary Table 2 and classified into five categories, symbolized by $V_{chunk\_category}$. Category 1 features a "1+1+1+1" structure where all four items are treated as separate entities. Category 2 encompasses the "2+1+1" structures, offering six possible configurations. Category 3 involves the "2+2" structures, presenting three possible configurations. Category 4 comprises the "3+1" structures, with four possible configurations. Lastly, category 5 represents the structure where all four items are treated as a single unit. The number of chunks for these 5 categories are represented by $V_{num\_of\_chunk\_in\_category}$. The assignment of items to chunks is represented by $V_{chunk\_item\_assignment}$, which is a 15 rows × 4 columns matrix. For instance, in chunking structure 3, the four numbers 1, 2, 1, and 3 imply that items 1 and 3 belong to a two-item chunk (chunk 1), while items 2 and 4 each belong to their own single-item chunks (chunks 2 and 3 respectively). All this information is outlined in Supplementary Table 2.

After defining these variables, the within-chunk variability ($V_{within\_chunk\_variability}$) and between-chunk variability ($V_{between\_chunk\_variability}$) are calculated as the sum of color differences ($V_{color\_difference}$) of all pairs of items that reside within the same chunk or between two chunks, taking into account $V_{interaction\_on\_bias}$. Next, the strength of a chunking structure ($V_{chunk\_structure\_strength}$) is calculated as the difference between $V_{between\_chunk\_variability}$ and $V_{within\_chunk\_variability}$. Conceptually, if items are similar within the same chunk and dissimilar otherwise, that denotes an appealing chunking structure.

The likelihood of all chunking structures ($V_{chunk\_structure\_likelihood}$) is then computed as the exponential value of $V_{chunk\_structure\_strength}$, modulated by a scaling factor of $P_{utility\_of\_chunk\_structure\_strength}$.

The chunking effect ($V_{chunking\_effect}$) is calculated as a weighted average across all 15 possible chunking structures, with $V_{chunk\_structure\_likelihood}$ serving as the weights. For each structure, the chunking effect is principally determined by the "reduction in the number of storage units" (4 - $V_{num\_of\_chunk\_in\_category}$). For instance, a category 2 structure contributes 1 unit of chunking effect because it reduces 4 items to 3 chunks, while a category 5 structure contributes 3 units due to a reduction from 4 items to 1 chunk.

Chunking structure	Category ($V_{chunk\_category}$)	Nature of chunks	Number of chunks ($V_{num\_of\_chunk\_in\_category}$)	Chunking effect	Assignment for each item ($V_{chunk\_item\_assignment}$)
1	1	1+1+1+1	4	0	1 2 3 4
2	2	2+1+1	3	1	1 1 2 3
3					1 2 1 3
4					1 2 3 1
5					2 1 1 3
6					2 1 3 1
7					2 3 1 1
8	3	2+2	2	2	1 1 2 2
9					1 2 1 2
10					1 2 2 1
11	4	3+1	2	2	2 1 1 1
12					1 2 1 1
13					1 1 2 1
14					1 1 1 2
15	5	4	1	3	1 1 1 1

Supplementary Table 2. All 15 possible chunking structures. They are divided into 5 categories. See text for details.

Step 2a. Eight color categories

As depicted in Figure 7a of the main text, this model defines eight color categories, each following a normal distribution. The centers, SDs, and amplitudes of these distributions are defined by $P_{center\_of\_8\_color\_category}$, $P_{log\_SD\_of\_8\_color\_category}$, and $P_{log\_amplitude\_of\_8\_color\_category}$, respectively, with the latter two parameters expressed in logarithmic values for convenience. Notably, each array possesses only seven freely varying parameters since the eighth category (red 2) is deliberately rendered identical to the first category (red) in all three dimensions.

As explained in the subsequent Step 3a, each color-category-biased component leans towards the center of its respective category. The degrees of attraction for the 8 categories are defined by $P_{log\_attraction\_of\_8\_color\_category}$, which is also expressed in logarithmic terms.

These elements — the SDs, amplitudes, and degrees of attraction — are all modulated by unequal spatial attention (see Supplementary Discussion 3.1). This phenomenon, denoted by $V_{spatial\_attention\_disadvantage}$, also plays a vital role in two subsequent steps: step 2c and 3d. Regarding the specific unequal distribution of spatial attention (see Figure 5f of the main text), the top-left item has a distinct advantage over the bottom two items, while the top-right item occupies a middle ground.

The magnitude of the effect of unequal spatial attention on these variables is specified by $P_{spatial\_attention\_on\_variable}$. Specifically, better-attended items' color categories are narrower (i.e., smaller SDs) and taller (i.e., greater amplitudes), and are less effective at attracting the color-category biased component (i.e., exhibit lesser degrees of attraction). Furthermore, the magnitude of the spatial attention effect for the eight color categories ($V_{spatial\_attention\_for\_category}$) is calculated as the exponential of $-P_{log\_attraction\_of\_8\_color\_category}$.

Once the effects of unequal spatial attention are taken into account, the SDs, amplitudes, and the degrees of attractions are respectively represented by $V_{SD\_of\_8\_color\_category\_X\_4\_item}$, $V_{amplitude\_of\_8\_color\_category\_X\_4\_item}$, and $V_{attraction\_of\_8\_color\_category\_X\_4\_item}$. Each of these variables is a matrix with 8 rows (representing the 8 categories) and 4 columns (representing the 4 items).

Step 2b. Weights of color-category-biased components

Here, for each of the 40,000 items, eight weights are calculated and recorded in columns 1 through 8 of $V_{weight\_for\_component}$. Each weight corresponds to one of the eight color-category-biased components and is determined by the distribution values of the eight categories (see Figure 7a of the main text) in relation to that item's color, which also considers the influence of $V_{interaction\_on\_bias}$. The SD and amplitude of the eight categories are determined by $V_{SD\_of\_8\_color\_category\_X\_4\_item}$ and $V_{amplitude\_of\_8\_color\_category\_X\_4\_item}$, respectively, both of which originate from step 2a.

Step 2c. Weights of unbiased component

Here, the weights of the unbiased component are computed and recorded in the 9th column of $V_{weight\_for\_component}$. These weights are influenced by $V_{spatial\_attention\_disadvantage}$ (see step 2a), which is scaled by the factor $P_{spatial\_attention\_on\_unbiased}$.

Step 2d. Concentration

The concentration effect, represented as $V_{concentration\_effect}$, is a mechanism in which the weights of items within a pattern influence one another. Specifically, the impact of an "affecting item" on an "affected item" operates in this manner: the weight of the affected item ($V_{weight\_affected\_item}$) decreases based on a proportion calculated as follows:

$$\log\left(\frac{Weight_{affecting\_item}}{Weight_{affected\_item}}\right)$$

Essentially, this results in a concentration effect, where a larger proportion of weight decrement is applied to the affected item when its weight is significantly overshadowed by that of the affecting item.

The concentration effect is modulated by the color difference between the affecting and affected items. This modulation is defined by the function $V_{concentration\_function}$ (see Figure 5c of the main text), the magnitude of which is determined by $P_{concentration\_magnitude}$, and the SD by $P_{general\_SD}$ (see step 1a).

Upon incorporating the concentration effect, the weights can, albeit infrequently, drop to negative values. These negative values are not applicable for subsequent computations and are therefore adjusted to a floor value of zero.

Step 2e. Crosstalk

Like the concentration effect, the crosstalk effect ($V_{crosstalk\_effect}$) is another mechanism where the weights of items within a pattern influence each other. Specifically, the magnitude of the crosstalk effect is determined solely by the weight of the affecting item ($V_{weight\_affecting\_item}$). At its core, this represents a "crosstalk" process in which a certain proportion of the weight is redistributed to other items.

The crosstalk effect is modulated by the color difference between the affecting and affected items. This modulation is defined by the function $V_{crosstalk\_function}$, which is composed of a positive short-range component and a negative long-range component (see Figure 5d of the main text). The latter is centered at 180°, which is the opposite side of the color wheel. The magnitudes of both components are determined by the parameter $P_{crosstalk\_magnitude}$. The SDs of the short-range and long-range components are specified respectively by $P_{crosstalk\_SD}$ and $P_{general\_SD}$ (see step 1a).

Upon incorporating the crosstalk effects, the weights can, albeit infrequently, drop to negative values. These negative values are not applicable for subsequent computations and are therefore adjusted to a floor value of zero.

Step 2f. Weights of Swap-based components

In this step, the weights of the swap-based components are computed, and the resulting values are saved in columns 10 through 12 of $V_{weight\_for\_component}$. Each weight is a function of $V_{color\_difference}$, taking the form of a normal distribution centered at zero. The SD and amplitude of this normal distribution are denoted by $P_{swap\_weight\_SD}$ and $P_{swap\_weight\_amplitude}$, respectively. Put simply, swaps occur only between similar items.

The weights of the swap-based components are also influenced by $V_{chunking\_effect}$ (from step 1b) and modulated by a scaling factor of $P_{chunking\_on\_swap}$. Specifically, swaps are less likely to occur within better-chunked patterns.

Step 2g. Weights of all 3 types of components

In this step, $V_{weight\_for\_component}$, which comprises the weights of color-category-biased components (columns 1-8), the weight of the unbiased component (column 9), and the weights of swap-based components (columns 10-12), is normalized and stored in the variable $V_{normalized\_weight\_for\_component}$.

Step 3a. Attraction toward centers

In this model, every color-category-biased component is attracted toward the center of its corresponding category. The degrees of this attraction, spanning 40,000 items × 8 components, is represented by $V_{degree\_of\_attraction\_toward\_category\_center}$. This variable is primarily determined by $V_{attraction\_of\_8\_color\_category\_X\_4\_item}$ (from step 2a). It is also affected by $V_{chunking\_effect}$ (from step 1b) and modulated by a scaling factor denoted by $P_{chunking\_on\_attraction}$. Specifically, better-chunked patterns are less attracted toward category centers.

Step 3b. Biases of distributions

In this step, biases for each of the 12 components across the 40,000 items are calculated, and these biases are stored in $V_{bias\_of\_all\_component}$.

Columns 1-8 of $V_{bias\_of\_all\_component}$ denote the biases of color-category-biased components ($V_{bias\_of\_category\_biased\_components}$). These biases are computed as the product of $V_{displacement\_from\_category\_center\_to\_item\_color}$ and $V_{degree\_of\_attraction\_toward\_category\_center}$ (from step 3a). Conceptually, if $V_{degree\_of\_attraction\_toward\_category\_center}$ is 0, the biased center (of the distribution for this color-category-biased component) remains on the item color. If it's 1, the biased center aligns with the category center, and if it's 0.5, the biased center is positioned midway between the item color and the category center.

Column 9 of $V_{bias\_of\_all\_component}$ represents the bias of the unbiased component, which, by definition, is always zero.

Columns 10-12 of $V_{bias\_of\_all\_component}$ represent the biases of swap-based components ($V_{bias\_of\_swap\_based\_components}$), which, by definition, are the color differences ($V_{color\_difference}$) between the swapped items and the target item.

Finally, $V_{interaction\_on\_bias}$, calculated in step 1a, is added to $V_{bias\_of\_all\_component}$.

Step 3c. SDs of distributions

In this step, SDs for each of the 12 components across the 40,000 items are calculated and stored in $V_{SD\_of\_all\_component}$.

Columns 1-8 of $V_{SD\_of\_all\_component}$ represent the SDs of color-category-biased components ($V_{SD\_of\_category\_biased\_component}$). These are proportionate to the SDs of the weights (i.e., categories) themselves, and are calculated by multiplying $P_{SD\_of\_8\_color\_category\_X\_4\_item}$ (from step 2a) by a constant ratio ($P_{SD\_ratio\_component\_to\_weight}$ = 80.8%). However, an exception occurs with the color-category-biased component associated with the "red 2" category: its SD is only 0.452 (specified by $P_{red\_advantage\_as\_category}$) of what is predicted by this relationship.

Column 9 of $V_{SD\_of\_all\_component}$ represents the SDs of the unbiased component ($V_{SD\_of\_unbiased\_component}$). These SDs are larger (i.e., less precise) for reddish colors than for other colors. In logarithmic values, the baseline SD of the unbiased component is specified by $P_{log\_SD\_unbiased\_baseline}$, and the magnitude of the increment in SD (i.e., reduction in precision) for reddish colors is specified by $P_{red\_disadvantage\_on\_unbiased}$.

Columns 10-12 of $V_{SD\_of\_all\_component}$ represent the SDs of the swap-based components ($V_{SD\_of\_swap\_based\_components}$). Similar to the color-category-biased components, these SDs ($V_{swap\_component\_SD}$) are proportionate to the SDs of the distributions for the weights of these components ($V_{swap\_weight\_SD}$ from step 2f), and are calculated by multiplying the latter by the same constant ratio ($P_{SD\_ratio\_component\_to\_weight}$ = 80.8%).

Step 3d. Retention rates of items

In this step, retention rates for the 40,000 items are calculated and stored in $V_{z\_retention}$. However, it's important to note that $V_{z\_retention}$ represents the Z-scores of the retention rates, not the rates themselves. The $V_{z\_retention}$ is primarily calculated as the sum of the weighted average of the distributions of the eight categories on the color wheel ($V_{the\_8\_category\_on\_color\_wheel}$), plus a constant baseline ($P_{retention\_baseline}$). For the former, the weights of these categories ($V_{weight\_for\_retention}$) are determined through a weighted average of the SDs (exponentials of $V_{log\_SD\_of\_8\_color\_category}$) and degrees of attraction (exponentials of $V_{log\_attraction\_of\_8\_color\_category}$) pertaining to each category. The necessary parameters for these calculations are specified by $P_{retention\_weight\_p}$.

$V_{z\_retention}$ is additionally influenced by $V_{interaction\_on\_retention}$ and $V_{spatial\_attention\_disadvantage}$, stemming from steps 1a and 2a, respectively. The magnitude of the second effect is determined by $P_{spatial\_attention\_on\_retention}$.

Step 3e. Trade-off

In this step, a trade-off takes place between the quantity (represented by retention rates) and the quality (represented by SDs) of VWM representations. $V_{normalized\_z\_retention}$ and $V_{normalized\_SD}$ are computed to evaluate the relative position of the retention rates and SDs for a pattern in comparison to all 10,000 patterns. Then, these two aspects mutually influence each other: $V_{z\_retention}$ is affected by $V_{normalized\_SD}$ and $V_{SD\_of\_all\_component}$ is influenced by $V_{normalized\_z\_retention}$, both at a rate specified by $P_{trade\_off\_effect}$.

$V_{normalized\_SD}$ is both calculated and adjusted in logarithmic scale to enhance linearity. Furthermore, $V_{normalized\_SD}$ determines an item's SD as a weighted average of the SDs of all its 12 components.

Step 3f. Distribution of responses (with low-precision components)

In this step, the response distributions for the 40,000 items are computed and stored in $V_{distribution\_of\_responses}$ as follows:

Initiating the process involves calculating the response distribution for each of the 12 components of every item. These components are modeled as normal distributions, with their centers and SDs determined by $V_{bias\_of\_all\_component}$ from Step 3b and $V_{SD\_of\_all\_component}$ from Step 3c. Then, the 12 components of each item are merged into a single entity, $V_{response\_sum}$, using the weights from $V_{normalized\_weight\_for\_component}$ in step 2g.

Subsequently, the $V_{response\_sum}$ of the usual-precision component was merged with that of the low-precision component to form $V_{response\_sum\_with\_low\_precision}$. The SD of the low-precision component is 4.56

times that of the usual-precision component, as specified by $P_{low\_precision\_SD\_ratio}$, while the fraction pertaining to the low-precision component is 4.5%, as specified by $P_{low\_precision\_portion}$.

Occasionally, $V_{response\_sum\_with\_low\_precision}$ drop to negative values[3]. These negative values are unsuitable for subsequent calculations and are therefore adjusted to a minimum value of zero. The result of this correction is then stored in $V_{knowledge\_based\_responses}$, which is normalized before further processing.

Finally, $V_{distribution\_of\_responses}$ is calculated as a blend of knowledge-based responses ($V_{knowledge\_based\_responses}$) and random guesses, the latter being characterized by a uniform distribution across the color wheel. The proportion of knowledge-based responses is determined using $V_{retention\_rate}$, which is calculated as the normcdf of the aforementioned $V_{z\_retention}$ from Step 3d.

Supplementary Discussion 2. Statistical evidence for the QCE-VWM model

Supplementary Discussion 2.1. The 17 alternative models

In the factorial comparison analysis by Van den Berg et al. (2014), the space of all 4×4×2=32 possible models was exhaustively tested to determine the globally optimal model. This approach is clearly impractical for this study. The current QCE-VWM model has 57 parameters, and exhaustively testing all possibilities for dropping a subset of these parameters would result in 2^{57} = 1.4E+17 models, not to mention the many other parameters that could have been included. This is why, as mentioned in the main text, the model development in this study diverges from the exhaustive testing typically employed in experimental psychology, opting instead for an iterative search approach common in AI research.

In this context, a reduced demonstration of optimality is adopted, showing that the QCE-VWM model is "optimal among the immediately conceivable alternatives." As discussed in the main text, the QCE-VWM model was compared to 17 alternative models. These models are listed in Supplementary Table 3. Fourteen of these models (models 2-15) were derived by eliminating a single mechanism or aspect, and they are used to show that each of these 14 mechanisms/aspects is essential for the QCE-VWM model. The remaining three (models 16-18) were developed by applying different methods to specific aspects of the QCE-VWM model, and they are used to show that each of these alternative methods is inferior to what is used in QCE-VWM.

To see the outcome of an alternative model, run the "show_model_results.m" script with the corresponding "i_model" from Supplementary Table 3.

[3] This occurs because the weights allocated to the swap-based components in Step 2f can sometimes become negative due to the chunking effect. Eliminating these negative values by the end of Step 2f impairs the model's performance. Conceptually, these negative weights seem to indicate a strategy of swap-based avoidance: in well-chunked patterns, the color values of other items are intentionally avoided. This needs to be further explored in the future.

Num		Model	NLLsr	t-tests for the 17 comparisons between the QCE-VWM and the 17 alternative models					See also
				t-value	p-value	Cohen's d	Δ_{param}	CAD	
1		QCE-VWM (N_{param} = 57)	5.073318						7
2	Eliminating	interactions between items	5.078633	83.1	<1.E-300	0.831	4	0.415	3.9
3	Eliminating	chunking	5.074214	51.8	<1.E-300	0.518	3	0.299	3.9
4	Eliminating	two categories	5.078256	120.6	<1.E-300	1.206	8	0.427	3.4
5	Eliminating	one category	5.074972	63.6	<1.E-300	0.636	4	0.318	3.4
6	Eliminating	red advantage	5.074454	67.0	<1.E-300	0.670	1	0.670	3.5
7	Eliminating	unbiased component	5.074289	59.2	<1.E-300	0.592	3	0.342	3.7
8	Eliminating	swap-based component	5.074367	51.0	<1.E-300	0.510	3	0.294	3.8
9	Eliminating	concentration	5.073677	24.8	2.94E-132	0.248	1	0.248	3.9
10	Eliminating	crosstalk	5.073902	36.9	2.97E-280	0.369	2	0.261	3.9
11	Eliminating	random guess	5.076610	89.9	<1.E-300	0.899	7	0.340	3.3
12	Eliminating	trade-off	5.073531	23.8	3.52E-122	0.238	1	0.238	3.3
13	Eliminating	low precision component	5.073609	37.8	2.12E-292	0.378	2	0.267	3.2
14	Eliminating	red disadvantage	5.073538	34.2	6.09E-243	0.342	1	0.342	3.5
15	Eliminating	unequal spatial attention	5.076726	101.5	<1.E-300	1.015	4	0.507	3.1
16	Applying	strict Bayesian rule	5.076897	108.5	<1.E-300	1.085	7	0.410	3.6
17	Applying	category-based difference	5.075305	51.3	<1.E-300	0.513	0	N/A	3.7
18	Applying	von Mises distribution	5.073461	20.1	6.55E-88	0.201	0	N/A	3.10

Supplementary Table 3. Model comparison. The NLLsr values for QCE-VWM and the 17 alternative models are listed in this table. In the 17 alternative models (models 2–18), fourteen of them (models 2-15) were derived by eliminating a single mechanism or aspect, while the remaining three (models 16-18) were developed by applying different methods to specific aspects of the QCE-VWM model. The QCE-VWM model consistently outperforms all 17 alternative models across the 10,000 patterns. In 17 two-tailed t-tests, the t-values, p-values, Cohen's d values, and CAD values (complexity-adjusted d, see Supplementary Methods 5.1 for details) are presented in this table. Many of the p-values are smaller than the smallest positive number in double-precision floating-point format so they are simply displayed as "<1.E-300". The Cohen's d values and CAD values presented here correspond to the green and purple bars, respectively, in Figure 4 of the main text. The rightmost "see also" column guides readers to the corresponding sections in the Supplementary Discussion where each model is elaborated.

Supplementary Discussion 2.2. Scripts of the alternative models

The specific ways in which the scripts for these 18 alternative models were created, by revising the script "QCE_VWM.m", are described in this section. After these revisions, each of the 18 models was optimized to determine its unique set of parameters, which were saved in the "saved_parameters.m" script.

The scripts for models 2 and 3 were developed by removing one step (step 1a for model 2; step 1b for model 3), except for the definition of those variables that would be used in subsequent steps.

The scripts for models 4 and 5 were developed by the removal of some elements (the 4th and 6th elements for model 4; only the 6th element for model 5) from $P_{center\_of\_8\_color\_category}$, $P_{log\_SD\_of\_8\_color\_category}$, $P_{log\_amplitude\_of\_8\_color\_category}$, and $P_{log\_attraction\_of\_8\_color\_category}$.

The scripts for models 6, 13, 14, and 15 were developed by setting specific variables to 0: $P_{red\_advantage\_as\_category}$ in step 3c (model 6), $P_{low\_precision\_portion}$ in step 3f (model 13), $V_{red\_disadvantage\_on\_unbiased}$ in step 3c (model 14), and $V_{spatial\_attention\_disadvantage}$ in step 2a (model 15).

The scripts for models 7 and 8 were developed by setting certain columns of $V_{weight\_for\_component}$ (column 9 for model 7; columns 10-12 for model 8) to 0 in step 2g.

The scripts for models 9, 10, and 12 were developed by eliminating a step (step 2d for model 9; step 2e for model 10; and step 3e for model 12).

The script for model 11 was developed by omitting steps 3d and 3e, and assigning $V_{retention\_rate}$ as 1.

Model 16 adheres to strict Bayesian principles, enforcing the additive rule and requiring that the degrees of attraction conform to Bayesian predictions. The script for model 16 was crafted with the following specific modifications: utilizing the SDs of categories as priors' SDs, introducing the SD of data as a new parameter, and employing Bayesian principles to anticipate both the SDs of color-category-biased components (i.e., SDs of posteriors) and the degrees of attractions. To facilitate understanding of these complex changes, a document titled "QCE_VWM/Model_16_changes.docx" is provided, with the changes highlighted using "track changes" mode.

The script for model 17 was crafted with the following amendments: initially swapping the orders of "steps 1a and 1b" with "steps 2a and 2b," then defining a variable $V_{color\_category\_based\_difference}$ at the end of step 2b, and substituting it for $V_{color\_difference}$ in various places in steps 1a and 1b. To facilitate understanding of these complex changes, a document titled "QCE_VWM/Model_17_changes.docx" is provided, with the changes highlighted using "track changes" mode.

The script for model 18 was crafted by substituting the truncated normal distribution with the von Mises distribution ("circ_vmpdf.m", downloadable at https://github.com/circstat/circstat-matlab) throughout the script. Note that this von Mises function calculates in the scale of radians rather than degrees, necessitating conversions.

Supplementary Discussion 2.3. Model comparison

Each of the 17 aforementioned alternative models was optimized to determine its own minimum NLLsr, and the optimized parameters were saved in the "saved_parameters.m" script. The NLLsr values of these 17 alternative models were then compared with the NLLsr of the QCE-VWM model in 17 t-tests which analyze the consistency of the difference of NLLsr across 10,000 patterns.

A statistical index can analyze consistency either across individual participants or across patterns. Although the former is more commonly used, the latter is chosen in this study because the amount of data is highly unequal among participants. In contrast, the amount of data for each pattern, allocated through random assignment, varies within a reasonably consistent range.

As shown in Supplementary Table 3, all the t-values are fairly large, and the corresponding p-values are extremely small[4]. This implies that all mechanisms of the QCE-VWM model are crucial and statistically validated. One may point out that these p-values are unusually small due to the large sample size (10,000 patterns). Considering this issue, Cohen's d, as a measure of effect size, is used as the major statistical index in this study because it does not scale with the dataset's size.

Both the Cohen's d values[5] and the CADs (complexity-adjusted d, see Supplementary Methods 5.1 for its definition) of these 17 comparisons are listed in Supplementary Table 3. They respectively describe the overall importance of a mechanism, and the importance after adjusting for complexity of the mechanism. These are the values shown by the green and purple bars in Figure 4 of the main text. As can be seen, most of these d values are decently large, but several are only slightly above 0.2, which could be considered small effects. However, Cohen's d is typically used in situations where the experiments are tailor-made to highlight one specific mechanism/factor, whereas the randomly generated patterns in the present study are not. With this consideration, it seems fair to say that all these effects are decent in magnitude.

Supplementary Discussion 2.4. Cross-validation

To further validate the mechanisms of the QCE-VWM model, cross-validation was undertaken. Specifically, for each of the 18 models (namely, the QCE-VWM model and the 17 alternative models), the "cross_validation_optimization.m" script evenly divided the data into 10 subsets (e.g., patterns 1-1,000; 1,001-2,000, etc.). It then employed the "optimization_whole_set.m" function to optimize each of these 10 subsets individually, thereby generating 10 sub-models. Subsequently, the parameters of all these sub-models were saved in the "cross_validation_saved_parameters.m" script.

Subsequently, for each of the 18 models, the "cross_validation_results.m" script applies each of the 10 sub-models to both the training set (i.e., the subset that the training was based on) and the validation set (the other 9/10 data that the training was not based on). And, like above, t-tests were used to assess the QCE-VWM model's advantage (i.e., smaller NLLsr values) over the alternative models across 10,000 patterns for both training set and the validation set.

For a direct index to assess the generalizability from training to validation set, generalizability ratio is defined as the ratio between the "Cohen's d for validation set" and "Cohen's d for training set", a ratio of 100% indicates that the advantage is perfectly generalizable, whereas a rate of 0% indicates that the advantage is completely artificial and non-generalizable. The average generalizability ratio is 99.5 %

---

[4] In case it is not self-evident, many of the candidate mechanisms/aspects that were rejected because they did not meet the above-mentioned criterion (CAD > 0.2) would have been considered significant under the usual standard (e.g., $p < 0.001$).

[5] It is worth noting that these 17 differences are generally positively skewed, ranging from 0.08 to 4.40 (mean = 1.36). This implies that the Cohen's d values and CADs would generally be slightly higher if the skewness were corrected. However, for the current purpose, the primary goal of these indices is to measure the contribution of a mechanism to the overall fit, rather than to assess the significance of a mechanism. Therefore, it is preferable to leave them uncorrected.

(refer to Supplementary Table 4 for detailed values) for the 17 advantages in the comparisons between the QCE-VWM and the 17 alternative models, suggesting that they are perfectly generalizable.

Num		Model	Cohen's d values for the t-tests across the 17 comparisons between the QCE-VWM model and the 17 alternative models.			Generalizability ratio from training to validation (%)
			The whole dataset	Training set	Validation set	
2	Eliminating	interactions between items	0.8306	0.8369	0.8312	0.9933
3		chunking	0.5179	0.5190	0.5181	0.9983
4		two categories	1.2064	1.2099	1.2037	0.9949
5		one category	0.6359	0.6306	0.6270	0.9943
6		red advantage	0.6703	0.6707	0.6644	0.9906
7		unbiased component	0.5916	0.5902	0.5851	0.9914
8		swap-based component	0.5096	0.5111	0.5087	0.9953
9		concentration	0.2484	0.2461	0.2484	1.0094
10		crosstalk	0.3695	0.3685	0.3630	0.9851
11		random guess	0.8990	0.9005	0.8986	0.9979
12		trade-off	0.2384	0.2385	0.2341	0.9815
13		low precision component	0.3780	0.3771	0.3716	0.9855
14		red disadvantage	0.3423	0.3364	0.3406	1.0124
15		unequal spatial attention	1.0149	1.0137	1.0121	0.9985
16	Applying	strict Bayesian rule	1.0848	1.0876	1.0812	0.9941
17		category based difference	0.5129	0.5080	0.5036	0.9912
18		von Mises distribution	0.2007	0.1909	0.1911	1.0009

Supplementary Table 4. Cross validation. Following a cross-validation process where the models are trained on 1/10 of the data and validated on the remaining 9/10 (see Supplementary Discussion 2.4 for more details), t-tests are conducted to compare the QCE-VWM model with each of the 17 alternative models for both the training and validation sets. The Cohen's d values for the training and validation sets (corresponding to the red and blue bars in Figure 4 of the main text) are provided here. The Cohen's d values for the whole dataset (represented by green bars in Figure 4 of the main text), which are listed in Supplementary Table 3, are repeated here for easier comparison. A generalizability ratio is defined as the ratio between the Cohen's d value for the validation set and the Cohen's d value for the training set and is also provided here. The average generalizability ratio for the 17 comparisons is 99.5%, suggesting perfect generalizability.

Supplementary Discussion 3. Other analysis and discussion

Supplementary Discussion 3.1. Spatial attention

As discussed in the main text, the QCE-VWM model demonstrated that VWM is spatially inhomogeneous. As illustrated in Figure 5f of the main text, the top-left item holds an advantage over the two bottom items, with the top-right item positioned in the middle. Specifically, the top-left item is

more likely to be remembered, i.e., it has a higher retention rate as implemented in step 3d. It is especially more likely to be remembered as the unbiased component, as implemented in step 2c. Its color categories are narrower and taller, and less effective at attracting the color-category biased component, as implemented in step 2a.

The model 15 eliminates spatial inhomogeneity and performs significantly worse than the QCE-VWM model (Cohen's d = 1.015, CAD = 0.507), underscoring the necessity of spatial inhomogeneity for the QCE-VWM model's effectiveness.

An important question arises regarding the cause of this spatial inhomogeneity effect. If, as speculated in the main text, the effect is caused by reading habits, then this implies that the spatial inhomogeneity effect is a manifestation of spatial attention. In other words, the locations may not be inherently unequal. Instead, they appear to be unequal because we are inclined to pay more attention to the top-left item than to the bottom items, with the top-right item falling in between.

Consistent with this hypothesis, it is well-documented that attention enhances the retention rate of visual working memory (VWM) items (e.g., see Schmidt et al, 2002), aligning with the findings presented here. Moreover, based on what we generally know about visual attention, it seems plausible that it would strengthen the "unbiased component" and also weaken the effect of color categories, again consistent with the current findings. Considering these points, the observed spatial inhomogeneity are tentatively interpreted as a "spatial attention" effect.

Admittedly, this attribution to spatial attention is speculative. It is possible that part of the spatial inhomogeneity may not be attributable to spatial attention but to other factors, such as asymmetry between the left-right and upper-lower visual fields. These possibilities need to be tested in future studies that directly manipulate spatial attention.

Supplementary Discussion 3.2. Low-precision component

There is a small proportion (4.5%) of a low-precision component in the QCE-VWM model. Model 13 eliminates this low-precision component and performs markedly worse than the QCE-VWM model (Cohen's d = 0.378, CAD = 0.267). This underscores the integral role that low-precision component play in the effectiveness of the QCE-VWM model. In other words, VWM comprises representations with varying precisions[6], rather than representations of a single precision.

---

[6] This low-precision component is designed to reflect the variability in precision previously reported (Fougnie, Suchow, & Alvarez, 2012; Van den Berg et al., 2012). However, it should be noted that the present study has greatly simplified its implementation. For example, Van den Berg et al. (2014) utilized a gamma distribution to characterize the distribution of precisions, whereas the present study added only a single low-precision component on the side of the primary normal-precision component. This simplification is necessary for practical reasons. Including a full range of variable-precision components would result in an optimization time much longer than what is currently experienced. As discussed in Supplementary Method 5.4, developing the current QCE-VWM model took eight months, making the additional cost unacceptable.

Supplementary Discussion 3.3. Slot vs. Resource

As discussed in the main text, the QCE-VWM model offers support for certain elements of both the slot and resource models.

On one hand, some aspects of the slot model indeed prove to be accurate. For instance, retention rates are affected by external factors such as spatial attention and interactions between items (step 3d), whereas the SDs of distributions are not (step 3c). Therefore, to a degree, the slot model correctly suggests that representation precision, but not retention rate, is a "fixed" attribute of VWM.

Moreover, even after considering other factors such as swaps, a significant proportion (averaging 14.7%) of responses appear to be random guesses, suggesting the presence of a real slot limit. Model 11, which seeks to eliminate the influence of random guesses[7], demonstrates significantly reduced data fitting performance (Cohen's d = 0.899, CAD = 0.340), thus highlighting the crucial role that these random guesses play in optimizing data fitting.

On the other hand, the resource model also presents valid points. Most notably, a trade-off between the quantity and quality of representations is observed in Step 3e, providing direct evidence for resource reallocation. Model 12 eliminates the trade-off mechanism, leading to a significantly compromised fitting performance (Cohen's d = 0.238, CAD = 0.238), thereby accentuating the pivotal role this trade-off serves in data fitting.

Furthermore, although the SDs of distributions (step 3c) are not affected by spatial attention and interactions between items, as mentioned earlier, they are influenced by factors such as color categories. This suggests a degree of flexibility in resource distribution. Additionally, as discussed above in Supplementary Discussion 3.2, VWM includes low-precision component, indicating that it is not limited to a single level of precision.

Overall, the QCE-VWM model provides a constructive resolution to the debate between the slot and resource models, integrating and evaluating insights from both within a single framework.

Supplementary Discussion 3.4. Color categories

As illustrated in Figure 7a of the main text, the QCE-VWM model incorporates eight color categories. All of them are indispensable. When the number of categories is reduced from eight to six (as seen in model 4) or to seven (as seen in model 5), the data fitting deteriorates significantly (model 4: Cohen's d = 1.206, CAD = 0.427; model 5: Cohen's d = 0.636, CAD = 0.318).

---

[7] It has been observed that the SD of the swap-based component has increased to the unrealistic value of 387.5° in model 11, compared to 23.4° in the QCE-VWM model. In other words, model 11 appears to have been forced to mimic random guesses through the swap-based component. This does not undermine the current findings because it merely suggests that random guesses are even more indispensable than demonstrated here. Future studies should be conducted to distinguish between random guesses and very imprecise responses.

It is important to note that the color categories depicted in Figure 7a of the main text likely depend on the specific color wheel. The amplitude and SD of these category distributions could vary significantly if a different color wheel is selected. Consequently, the critical insights gleaned from these color categories are not the specific characteristics of the categories themselves, but the discerned rules governing their influence on VWM. While the former may not apply to other potential selections of color wheels, it is hoped that the latter will.

Supplementary Discussion 3.5. Advantage and disadvantage of reddish colors

As illustrated in Figure 7b of the main text, the “Red 2” category emerges as a clear outlier among the eight categories. The SD of its corresponding color-category-biased component is notably smaller (i.e., more precise) than what would be anticipated based on the proportional relationship observed in the other seven categories. In this study, this unusually high precision for the color-category-biased-component of reddish colors is referred to as the “red advantage.” The necessity of the red advantage in the QCE-VWM model is confirmed by the results from model 6. In this model, the SD of the color-category-biased component for the “Red 2” category is constrained to follow the proportional relationship, leading to a significant deterioration in the model’s performance (Cohen’s d = 0.670, CAD = 0.670).

The mean, SD, and amplitude of the “Red 2” category are all identical to those of the “Red” category. Therefore, although they are modeled and referred to as two categories for convenience, they probably are not two distinct color categories but rather different functionalities of a single category. Specifically, the “Red” category represents the general functionality that applies to all color categories, while the “Red 2” category represents a unique functionality specific to reddish colors, allowing them to be memorized with unusually high precision.

On the other hand, as demonstrated in Figure 7c of the main text, there is a “Red disadvantage.” Specifically, for the unbiased component, reddish colors are much less precise than other colors. The necessity of acknowledging the red disadvantage within the QCE-VWM model is corroborated by the findings from model 14. In this model, the SD or precision of the unbiased component is constrained to remain constant across the color wheel, resulting in a notable decline in the model’s performance (Cohen’s d = 0.342, CAD = 0.342).

The question of why reddish colors appear more precise in one case but less precise in another may seem contradictory at first, but it could actually be two aspects of the same phenomenon. The color wheel used in the current study is designed to be approximately perceptually uniform (refer to Supplementary Methods 1.2), indicating that the efficacy of color processing is constant around the wheel. To simplify, consider that this overall efficacy equals the sum of the efficacy of the color-category-biased component and the efficacy of the unbiased component. If the color-category-biased component is exceptionally strong due to a “red advantage,” then the unbiased component will conversely be weaker, resulting in a “red disadvantage.”

In other words, this “red disadvantage” does not necessarily imply an intrinsic flaw in reddish colors in the unbiased component but may simply indicate that their perceived strengths in the CIE-Lab color

space have been overstated due to the red advantage in the color-category-biased component. This situation mirrors the statistical concept known as “range restriction.” For instance, in educational contexts, admission policies that require a combined score from a mathematics test and a verbal test may lead to the artificial observation that students who are especially good in mathematics will tend to be poor in verbal skills, even if there is no genuine conflict between these aspects. Similarly, the fixed overall level of color processing efficacy in this study means that a red advantage in one aspect inevitably causes a red disadvantage in another.

Supplementary Discussion 3.6. Deviations from Bayesian principles

The color-category-biased components gravitate towards the category centers and are therefore Bayesian-like mechanisms. However, they fundamentally differ from strict Bayesian principles in two respects.

First, according to Bayesian principles, there should be an additive relationship between the precision of the posterior (i.e., color-category-biased components) and the precision of the prior (i.e., their respective categories); the former should equal the sum of the latter and the precision of the pre-Bayesian data. However, as illustrated in Figure 7b of the main text, a multiplicative relationship, wherein the former is proportional to the latter, aligns more accurately with the data.

Second, Bayesian principles dictate that the degree of attraction towards a category center should equal the precision of the prior divided by the precision of the posterior. Yet, as depicted in Supplementary Figure 3, the actual degrees of attraction do not conform to Bayesian predictions.

Model 16, which implements strict Bayesian principles, exhibits significantly deteriorated performance (Cohen’s d = 1.085, CAD = 0.410), implying that deviations from Bayesian principles are necessary.

The identification of these deviations from Bayesian principles shows that a comprehensive exploration can analyze cognitive mechanisms more precisely than usual-scale experiments can. In the current study, the QCE-VWM model significantly outperformed model 16. However, this advantage constitutes only a “minor difference” when compared to the disparity between possessing and not possessing an attraction-toward-category-center mechanism. Consequently, in a usual-scale experiment, the most likely interpretation would have been to highlight the superiority of the Bayesian model over the model without an attraction-toward-category-center feature, while ignoring any deviations from the Bayesian model. This might explain why such deviations have not been documented before. Even if they are present, it’s probable that they would not be reported in a usual-scale experiment.

The preceding discussion does not imply that these specific deviations are unequivocally correct. In fact, the degree of attraction seems to be a relatively weak aspect of the QCE-VWM model in terms of conceptual plausibility. For instance, as illustrated in Supplementary Figure 3, the degrees of attractions for two categories (red and cyan) exceed one, suggesting that these color-category-biased component are “excessively” attracted to the opposite side of the category, which is somewhat puzzling. Further studies are needed to determine whether a more elegant explanation will ultimately surface.

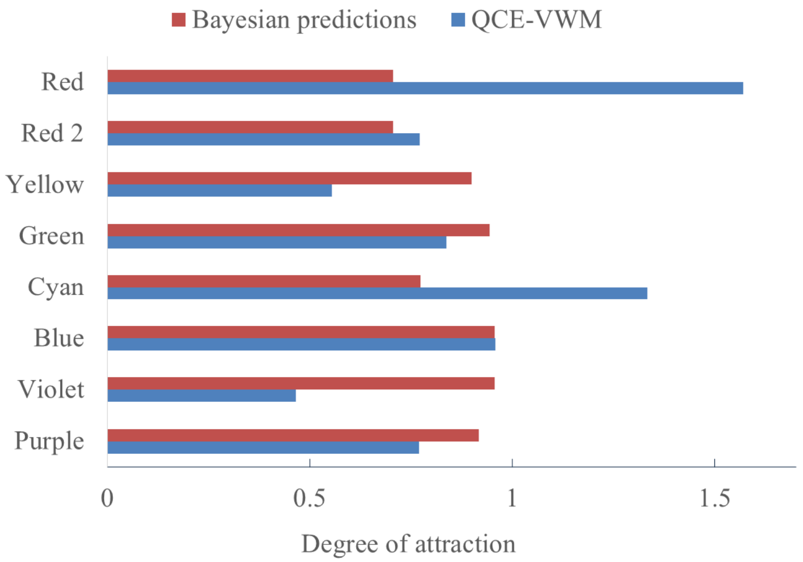

Supplementary Figure 3. The degree of attraction for the eight categories. The values in the QCE-VWM model (blue bars) differ greatly from those predictions made by Bayesian principles (red bars).

Supplementary Discussion 3.7. Early vs. late processing

The notions of early and late processing frequently appear in studies examining perceptual and cognitive processes. Although these terms may be somewhat ambiguous, a generally accepted distinction holds that early processing is based on perceptual input, whereas late processing is influenced by high-level factors such as strategy or experience. The structure of the QCE-VWM model aligns well with this distinction. In this task, color categories serve as the critical high-level factor distinguishing between the early and late phases of the model.

On one hand, the initial perceptual input dominates the early stage, with the high-level factor (color categories) playing no role. Specifically, the two early mechanisms — interactions between items and chunking — in phase 1 rely on pre-categorical color differences rather than category-based differences. To support this, when model 17 compels these two mechanisms to utilize category-based differences, its performance noticeably declines (Cohen's d = 0.513).

On the other hand, color categories become dominant in the late stage, reducing the initial perceptual input to a relatively minor role. This is demonstrated by comparing the contribution of the "unbiased component" to that of other components. Model 7, which eliminates the unbiased component, performs significantly worse than the QCE-VWM model (Cohen's d = 0.592, CAD = 0.342), underscoring the necessity of the unbiased component. It is hardly surprising, given that it represents the "real" color that should have been memorized. However, the role of the unbiased component is considerably smaller compared to the color-category-biased components. Its elimination results in an impact comparable to reducing the color-category-biased components by one (model 5: Cohen's d = 0.636, CAD = 0.318), and is far less consequential than reducing them by two (model 4: Cohen's d = 1.206, CAD = 0.427). To draw another comparison, the effect of removing the unbiased component is

only slightly larger than that of eliminating the swap-based components (model 8: Cohen's d = 0.510, CAD = 0.294), implying that the "real color" is not much more important than the "swap-based errors." In summary, it is clear that in the late stage, color categories take precedence, relegating the initial perceptual input to a subsidiary role.

Supplementary Discussion 3.8. Spatial binding errors

The spatial binding errors refer to situations in which observers correctly report the color of an item but attribute it to the wrong item (e.g., Bays, Catalao, & Husain, 2009). For example, a purple square is presented in the top-left corner of the display, but observers more frequently report a purplish color to the bottom-left corner than would be expected from random guessing.

Spatial binding errors have important implications for the aforementioned slot/resource debate because they make it difficult to distinguish between "knowledge-based but mislocalized responses" and random guesses. Therefore, they are modeled as one of the three aspects of factorial comparison analysis by van den Berg et al. (2014). In this regard, the present study suggests that both spatial binding errors and random guesses are real components of the VWM responses.

A further question concerns the nature of spatial binding errors. Do they occur in the representation stage or the response stage? The current study has considered both possibilities. The swap-based component in the QCE-VWM model is designed to reflect spatial binding errors at the representation stage. Specifically, the color value of one item replaces that of another. Model 8, which excludes these swap-based components, significantly underperforms (Cohen's d = 0.510, CAD = 0.294), implying that these components are indispensable for the QCE-VWM model. On the other hand, attempts to implement spatial binding errors at the response stage have not led to a large enough improvement in the model (i.e., CAD>0.2). To put it simply, spatial binding errors occur at the representation stage, not the response stage. In other words, the issue is that colors are genuinely remembered at the wrong location, not that they are remembered at the correct location but reported at the wrong one.

Supplementary Discussion 3.9. How items affect each other

In addition to swap-based responses, there are other possible ways in which items within the same pattern can affect one another. Step 1a of the QCE-VWM model addresses interactions between items and was developed based on findings from previous studies (Brady & Alvarez, 2011). The significance of this mechanism within the QCE-VWM model is substantiated by the diminished performance (Cohen's d = 0.831, CAD = 0.415) observed in model 2, which eliminated the interactions between items in step 1a.

Furthermore, chunking represents another classic mechanism through which items affect each other. The significance of the chunking mechanism within the QCE-VWM model is underscored by the diminished performance (Cohen's d = 0.518, CAD = 0.299) observed in model 3, which removed the chunking mechanism in step 1b.

An important distinction exists between "interactions between items" and "chunking." The former focuses on the effects at the individual item level (i.e., how interactions affect the representations of

individual items), which includes enhancing retention rates and introducing biases. In contrast, the latter concerns itself solely with effects at the whole-pattern level (i.e., how chunking, as a specific type of interaction, affects the pattern as a whole). Specifically, the effects of interactions between items typically vary for each individual item. In contrast, the chunking effect, as implemented in this study, is always a whole-pattern-level index that describes how "well-chunked" the pattern is. For example, in a "2+1+1" chunking structure, the benefit of chunking is equal across all four items, regardless of whether an item is part of the two-item chunk or is an isolated item.

Moreover, I have explored several other potential ways how items can affect each other. The only case where this has been confirmed is in the finding that the weights of the items influence one another. Specifically, this leads to the creation of concentration (step 2d) and crosstalk (step 2e) mechanisms. Both of these mechanisms are essential to the functionality of the QCE-VWM model, as evidenced by the significantly reduced data-fitting performance of model 9 (Cohen's d = 0.248, CAD = 0.248) and model 10 (Cohen's d = 0.369, CAD = 0.261), which had the concentration and crosstalk mechanisms removed, respectively.

These diverse lines of findings all underscore the importance of understanding the ways in which items affect each other. A critical question is whether it will be possible to find a unified explanation for all these mechanisms. Specifically, as discussed above, "interactions between items" and "chunking" focus respectively on individual-item-level and whole-pattern-level effects. It would be desirable to address these two levels of effects with a single mechanism. Indeed, several attempts have been made toward that goal, albeit without success. Future studies will be needed to determine whether a unified account of these two mechanisms, ideally also incorporating concentration and crosstalk, can be achieved.

Supplementary Discussion 3.10. Normal distribution in circular space

The normal distribution is one of the most commonly used probability distributions. However, the normal distribution cannot be directly applied to circular data, such as angles, because it is defined over a linear or unbounded space. In such situations, like many previous studies of working memory, the von Mises distribution is commonly used as a substitute for the normal distribution in circular space.

Despite the popularity of the von Mises distribution, other alternatives exist. The QCE-VWM model utilizes a "truncated normal distribution" in which the value at a point is determined by the shortest distance between that point and the distribution's center on the circle. In other words, this truncated normal distribution effectively results from wrapping a normal distribution around a circle and truncating the tails beyond the point where the distribution intersects itself[8]. The probability density

[8] Another possible cyclic variant of the normal distribution is the wrapped normal distribution, in which the values are wrapped around a circle. For instance, the pdf of this distribution at 0° accumulates the normal pdf at 0°, ±360°, ±720°, and so on, in a repetitive cycle. Although the idea of repetitive cycles makes sense in some other contexts (e.g., the memory of summer is accumulated from both the immediate past summer and all previous summers), it seems absurd to conceptualize the effects of color differences as a summation of repetitive cycles. Instead, what appears to matter most is the shortest

function (pdf) of the truncated normal distribution is implemented by a custom-made function in "truncated_normal_pdf.m." Because the tails are truncated, the total probability of the truncated normal distribution is slightly less than 1. Therefore, when using this distribution to model the distribution of responses, it is renormalized to ensure that its total probability integrates to 1.

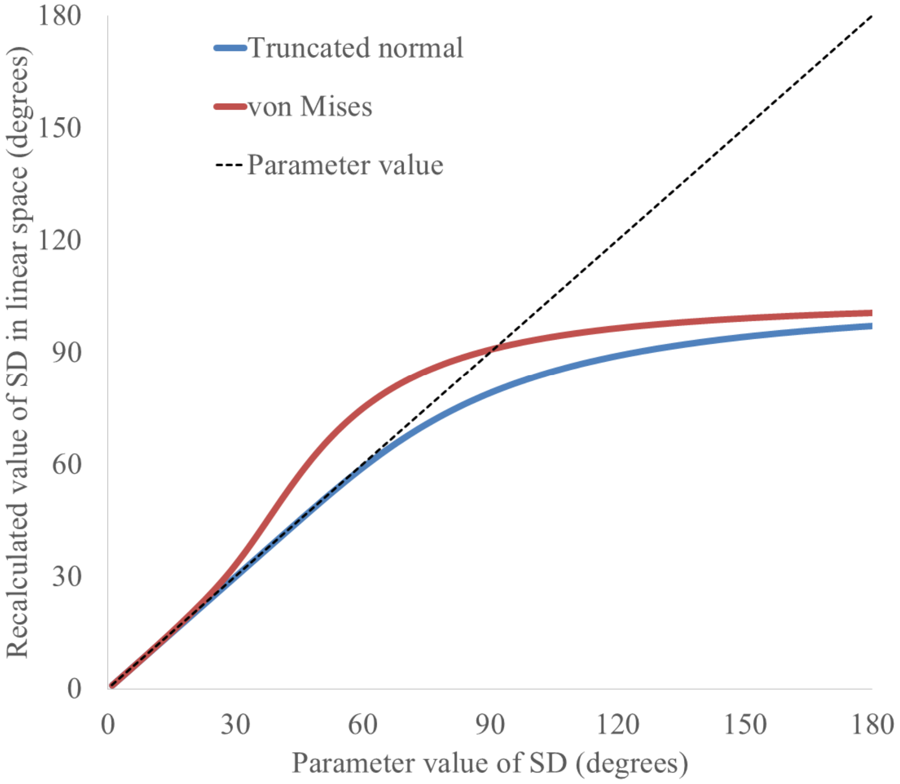

Supplementary Figure 4. Recalculated Value of SD. This graph illustrates how the recalculated value of SD varies with the parameter value of SD. Specifically, this recalculation treats the circular space distribution as if it resides within the [-180°, 180°] range of a linear space. Within the 30° to 60° precision range, the recalculated SD from the truncated normal distribution aligns with the parameter SD, but the recalculated SD from the von Mises distribution does not.

In model 18, the truncated normal distribution is replaced by the von Mises distribution. The performance of this model 18 significantly worsens (Cohen's d = 0.201), indicating that the truncated normal distribution, although not as popular as the von Mises distribution, better reflects the underlying mechanism of the VWM.

Why does the truncated normal distribution outperform the von Mises distribution? As illustrated in Supplementary Figure 4, The SD of the truncated normal distribution aligns more closely with the

distance on the color wheel, making the truncated normal distribution more suitable for this study. Empirically, the truncated normal distribution has been compared to the wrapped normal distribution, and they have produced very similar results, with a very slight advantage favoring the truncated normal distribution.

"recalculated SD" than the von Mises distribution does. This could explain why the truncated normal distribution more precisely mirrors the underlying mechanisms of VWM than the von Mises distribution does.

Supplementary Discussion 3.11. Order of report

Adam, Vogel, and Awh (2017) demonstrated that when observers are asked to memorize a few items and report them in any order they choose, the retention rate—though not the precision of memory representations—is significantly lower for later reports than for earlier ones. As discussed in Supplementary Methods 2.1, the present study focused on the analysis of pattern-level summaries; therefore, the QCE-VWM cannot incorporate the effect of the "order of report" because it is averaged out in the pattern-level summaries. Consequently, additional analysis has been conducted to assess the effect of the "order of report."

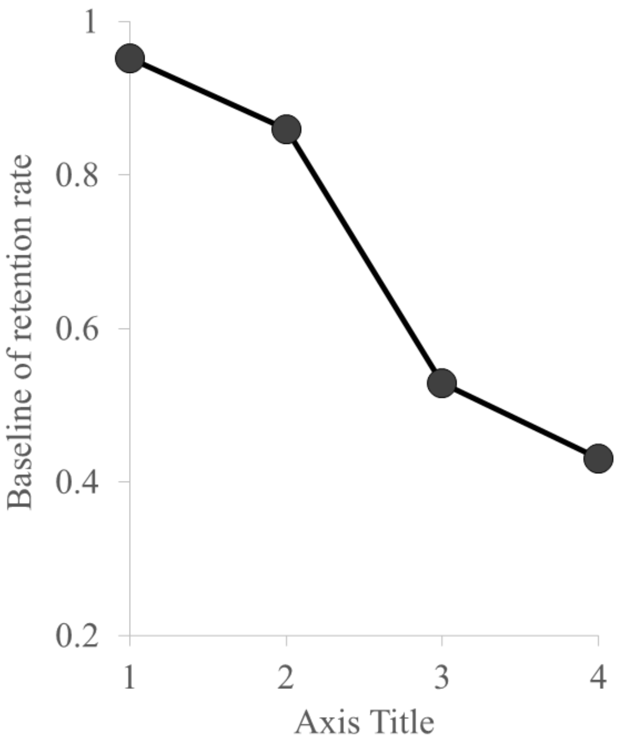

Supplementary Figure 5. Additional analysis on the order of report. Consistent with Adam, Vogel, and Awh (2017), retention rates are significantly lower for later reports than for earlier ones. This is the predominant effect of the order of report. Please note that the concerned parameter, $P_{retention\_baseline}$, directly affects the Z-score of the retention rates. Therefore, for easier understanding, this figure does not show the actual parameter values, but rather their normcdf conversions.

Here, potential effects of "order of report" are explored by identifying the parameters of QCE-VWM that are most crucial to the order of report. Specifically, the script "preliminary/formatted_data_to_distribution_order_of_report.m" divides the data into four separate "distributions of responses" for the first, second, third, and fourth reports of each trial and saves them in

"distribution_data_order_of_report.mat". Subsequently, the script "order_of_report_optimization" is used to optimize the QCE-VWM separately for each of the four distributions, enabling observation of how the parameters of the QCE-VWM vary depending on the order of reports. The optimized parameters are saved in "order_of_report_saved_parameters.m". Then, the script "order_of_report_show_results.m" calculates the cost of fixing a parameter across the four distributions and determines the cost for each of the 57 parameters individually. To clarify, the greater the cost of fixing a parameter, the more important it is to the order of report. Similar to the description in Supplementary Discussion 2.3, this cost is measured by the CAD.

The results of this analysis showed that one parameter, $P_{retention\_baseline}$, which specifies the general baseline level of the retention rate, is decisively more important than all other parameters (CAD = 0.779). In comparison, the second highest CAD is much lower (CAD = 0.333).

As shown in Supplementary Figure 5, the $P_{retention\_baseline}$ values are much lower for later reports than for earlier ones. In other words, the present study has confirmed the findings of Adam, Vogel, and Awh (2017) that retention rates are significantly lower for later than for earlier reports, and also that this is the single most important change depending on the order of report.

Supplementary Discussion 3.12. Age and gender

Similar to the effect of the "order of report" discussed in the preceding section, the effects of age and gender cannot be incorporated into the QCE-VWM because they are averaged out in the pattern-level summaries. Consequently, additional analyses have been conducted on these effects. Participants are divided into younger and older groups, with the cutoff point being that the younger group was born in 1993 or later. They are also divided according to self-reported genders (male vs. female). In total, the participants are divided into four groups.

In this section, the effects of age and gender are explored by identifying the parameters of the QCE-VWM that are most affected by these factors. Specifically, the script "preliminary/formatted_data_to_distribution_age_gender.m"[9] divides the data into four separate "distributions of responses" for the four groups (younger female, older female, younger male, older male) and saves them in "distribution_data_age_gender.mat". Subsequently, the script "age_gender_optimization" was used to optimize the QCE-VWM separately for each of the four distributions, enabling observation of how the parameters of the QCE-VWM vary depending on age and gender. The optimized parameters were saved in "age_gender_saved_parameters.m". Then, the script "age_gender_show_results.m" calculates the cost of fixing a parameter across the four distributions and determines the cost for each of the 57 parameters individually. To clarify, the greater the cost of fixing a parameter, the more important it is to the age and gender differences. Similar to the description in Supplementary Discussion 2.3, this cost is measured by the CAD.

---

[9] This script cannot run properly with the downloaded files because the user information file (user_info.xlsx) has been removed to protect individual users' privacy. If you wish to run this analysis, please contact Liqiang Huang at lqhuang@cuhk.edu.hk to obtain the file.

The results of this analysis showed that the largest CAD is merely 0.194. In other words, as far as this group of participants is concerned, the QCE-VWM generally applies to different age and gender groups, and none of the 57 parameters vary greatly across these groups.

References in the Supplementary Information

Adam, K. C., Vogel, E. K., & Awh, E. (2017). Clear evidence for item limits in visual working memory. *Cognitive Psychology, 97*, 79-97. doi:10.1016/j.cogpsych.2017.07.001

Agrawal, M., Peterson, J. C., & Griffiths, T. L. (2020). Scaling up psychology via scientific regret minimization. *Proceedings of the National Academy of Sciences, 117*(16), 8825-8835.

Bae, G.-Y., Olkkonen, M., Allred, S. R., & Flombaum, J. I. (2015). Why some colors appear more memorable than others: A model combining categories and particulars in color working memory. *Journal of Experimental Psychology: General, 144*(4), 744. doi:10.1037/xge0000076

Bays, P. M., Catalao, R. F., & Husain, M. (2009). The precision of visual working memory is set by allocation of a shared resource. *Journal of Vision, 9*(10), 7-7.

Brady, T. F., & Alvarez, G. A. (2011). Hierarchical Encoding in Visual Working Memory: Ensemble Statistics Bias Memory for Individual Items. *Psychological Science, 22*(3), 384-392. doi:10.1177/0956797610397956

Fougnie, D., Suchow, J. W., & Alvarez, G. A. (2012). Variability in the quality of visual working memory. *Nature Communications, 3*(1), 1229.

Schmidt, B. K., Vogel, E. K., Woodman, G. F., & Luck, S. J. (2002). Voluntary and automatic attentional control of visual working memory. *Perception & Psychophysics, 64*(5), 754-763. doi:Doi 10.3758/Bf03194742

Van den Berg, R., Awh, E., & Ma, W. J. (2014). Factorial comparison of working memory models. *Psychological Review, 121*(1), 124.

Van den Berg, R., Shin, H., Chou, W.-C., George, R., & Ma, W. J. (2012). Variability in encoding precision accounts for visual short-term memory limitations. *Proceedings of the National Academy of Sciences, 109*(22), 8780-8785.

Zhang, W. W., & Luck, S. J. (2008). Discrete fixed-resolution representations in visual working memory. *Nature, 453*(7192), 233-U213. doi:10.1038/nature06860
