# Supplementary material for: Comprehensive exploration of visual working memory mechanisms using large-scale behavioral experiment
Source: Nat Commun. 2025 Feb 5;16:1383. doi: 10.1038/s41467-025-56700-5 (PMC11799313; doi:10.1038/s41467-025-56700-5)
Supplement: Supplementary file 2 — Reporting Summary [file 41467_2025_56700_MOESM2_ESM.pdf]

nature portfolio

Corresponding author(s): Liqiang Huang

Last updated by author(s): Nov 27, 2024

Reporting Summary

Nature Portfolio wishes to improve the reproducibility of the work that we publish. This form provides structure for consistency and transparency in reporting. For further information on Nature Portfolio policies, see our Editorial Policies and the Editorial Policy Checklist.

Statistics

For all statistical analyses, confirm that the following items are present in the figure legend, table legend, main text, or Methods section.

n/a	Confirmed	
☐	☒	The exact sample size (*n*) for each experimental group/condition, given as a discrete number and unit of measurement
☐	☒	A statement on whether measurements were taken from distinct samples or whether the same sample was measured repeatedly
☐	☒	The statistical test(s) used AND whether they are one- or two-sided<br>*Only common tests should be described solely by name; describe more complex techniques in the Methods section.*
☒	☐	A description of all covariates tested
☐	☒	A description of any assumptions or corrections, such as tests of normality and adjustment for multiple comparisons
☐	☒	A full description of the statistical parameters including central tendency (e.g. means) or other basic estimates (e.g. regression coefficient) AND variation (e.g. standard deviation) or associated estimates of uncertainty (e.g. confidence intervals)
☐	☒	For null hypothesis testing, the test statistic (e.g. *F*, *t*, *r*) with confidence intervals, effect sizes, degrees of freedom and *P* value noted<br>*Give P values as exact values whenever suitable.*
☒	☐	For Bayesian analysis, information on the choice of priors and Markov chain Monte Carlo settings
☒	☐	For hierarchical and complex designs, identification of the appropriate level for tests and full reporting of outcomes
☐	☒	Estimates of effect sizes (e.g. Cohen's *d*, Pearson's *r*), indicating how they were calculated

Our web collection on statistics for biologists contains articles on many of the points above.

Software and code

Policy information about availability of computer code

Data collection	The data was collected online, and the computer code used to create the webpage for data collection was written in JavaScript, Vue.js (version 2.6.11), and PHP (version 5.3.3).
Data analysis	The QCE-VWM model was developed using MATLAB (R2022b), while the neural network was implemented using PyTorch version 1.12.0. All data and scripts used for the data analysis are publicly available on the Open Science Framework and can be accessed via the following link: https://doi.org/10.17605/OSF.IO/QPY49.

For manuscripts utilizing custom algorithms or software that are central to the research but not yet described in published literature, software must be made available to editors and reviewers. We strongly encourage code deposition in a community repository (e.g. GitHub). See the Nature Portfolio guidelines for submitting code & software for further information.

Data

Policy information about availability of data

All manuscripts must include a data availability statement. This statement should provide the following information, where applicable:

- Accession codes, unique identifiers, or web links for publicly available datasets
- A description of any restrictions on data availability
- For clinical datasets or third party data, please ensure that the statement adheres to our policy

All data and scripts used for the data analysis are publicly available on the Open Science Framework and can be accessed via the following link: https://doi.org/10.17605/OSF.IO/QPY49.

Research involving human participants, their data, or biological material

Policy information about studies with human participants or human data. See also policy information about sex, gender (identity/presentation), and sexual orientation and race, ethnicity and racism.

Reporting on sex and gender	This was an online experiment, and we only collected information on self-reported gender. Gender was not specifically considered in the design or execution of the experiments. According to the self-reported data, 59.3% of the participants identified as female. The effect of gender was analyzed (see Supplementary Information 9.12), and no significant differences were found.
Reporting on race, ethnicity, or other socially relevant groupings	This was an online experiment, and this information was not collected. However, the data collection platform was presented in Chinese and embedded within the WeChat app, suggesting that participants were probably Chinese language users.
Population characteristics	This online experiment was open to anyone, conducted as an online game that participants accessed using their personal devices, suggesting that most participants were likely active internet users. A total of 2,316 participants (59.3% female; mean age = 29.4) played the game. The data collection platform was presented in Chinese and embedded within the WeChat app, indicating that participants were likely Chinese language users. Individuals with color vision deficiencies were explicitly instructed not to participate. Beyond these factors, no other notable biases are apparent in the study population.
Recruitment	This online experiment was open to anyone and was conducted as an online game that participants accessed on their personal devices. It is likely that participants learned about the experiment through information shared among friends.
Ethics oversight	Ethical approval was obtained from the Research Ethics Committee of The Chinese University of Hong Kong prior to the commencement of the study (SBRE-19-224, approved on 6 February 2020; SBRE-21-0204, approved on 6 December 2021).

Note that full information on the approval of the study protocol must also be provided in the manuscript.

Field-specific reporting

Please select the one below that is the best fit for your research. If you are not sure, read the appropriate sections before making your selection.

☐ Life sciences ☒ Behavioural & social sciences ☐ Ecological, evolutionary & environmental sciences

For a reference copy of the document with all sections, see nature.com/documents/nr-reporting-summary-flat.pdf

Behavioural & social sciences study design

All studies must disclose on these points even when the disclosure is negative.

Study description	Quantitative large-scale experimental study
Research sample	This online experiment was open to anyone, conducted as an online game that participants accessed using their personal devices, suggesting that most participants were likely active internet users. A total of 2,316 participants (59.3% female; mean age = 29.4) played the game. The data collection platform was presented in Chinese and embedded within the WeChat app, indicating that participants were likely Chinese language users. Individuals with color vision deficiencies were explicitly instructed not to participate. Beyond these factors, no other notable biases are apparent in the study population.
Sampling strategy	This was an online experiment, and participation was open to anyone, making it a convenience sample. The target dataset size was determined based on an estimation of the total number of trials required. Specifically, based on previous experience with working memory studies, it was estimated that 1,000 trials would be sufficient for measuring VWM for each individual color pattern. Consequently, a total of 10 million trials was planned.
Data collection	The experiment was conducted as an online game that participants accessed using their personal devices.
Timing	June 2021 - June 2023

Data exclusions	Both poor blocks and unusually good blocks were excluded according to pre-determined criteria. See details in the "methods" section.
Non-participation	The present experiment was presented as an Online game. Probably, some people have seen the game and choose not to play it.
Randomization	A complete randomization was implemented on the level of trials. The participants were not divided into groups.

Reporting for specific materials, systems and methods

We require information from authors about some types of materials, experimental systems and methods used in many studies. Here, indicate whether each material, system or method listed is relevant to your study. If you are not sure if a list item applies to your research, read the appropriate section before selecting a response.

Materials & experimental systems

n/a	Involved in the study	
☒	☐	Antibodies
☒	☐	Eukaryotic cell lines
☒	☐	Palaeontology and archaeology
☒	☐	Animals and other organisms
☒	☐	Clinical data
☒	☐	Dual use research of concern
☒	☐	Plants

Methods

n/a	Involved in the study	
☒	☐	ChIP-seq
☒	☐	Flow cytometry
☒	☐	MRI-based neuroimaging

Plants

Seed stocks	NA
Novel plant genotypes	NA
Authentication	NA
